# Nanoemulgels as Advanced Topical Drug Delivery Systems: Mechanistic Insights and Therapeutic Applications in Skin Disorders, Infections, Wound Healing, and Cancer

**DOI:** 10.3390/ph19020247

**Published:** 2026-01-31

**Authors:** Shery Jacob, Anroop B. Nair

**Affiliations:** 1 Department of Pharmaceutical Sciences, College of Pharmacy, Gulf Medical University, Ajman 4184, United Arab Emirates; 2 Department of Pharmaceutical Sciences, College of Clinical Pharmacy, King Faisal University, Al-Ahsa 31982, Saudi Arabia; anair@kfu.edu.sa

**Keywords:** nanoemulsion, nanoemulgel, topical, preparation, wound healing, skin cancer, evaluation, patents

## Abstract

Nanoemulgels have emerged as a promising hybrid drug delivery system that integrates the advantages of nanoemulsions and gels, offering enhanced drug penetration, prolonged residence time, and improved patient compliance. This review provides a comprehensive overview of the therapeutic applications of nanoemulgels in wound healing, microbial infections, skin cancer, and various dermatological disorders. The article begins with an overview of skin architecture and its implications for cutaneous drug delivery, followed by a clear distinction between transdermal and topical drug delivery systems. The mechanisms of drug transport into and through the skin are discussed in detail, highlighting the role of nano-sized carriers, particularly nanoemulsions, in overcoming the stratum corneum barrier. Mechanistic insights into nanocarrier-mediated cutaneous drug transport and their versatility as dermal delivery platforms are described. The formulation aspects of nanoemulgels, including their components and both high-energy and low-energy methods for nanoemulsion preparation, are critically discussed to elucidate their impact on formulation performance. An overview of in vitro characterization techniques and biological screening methods employed to evaluate nanoemulgel performance is presented, along with a tabulated compilation of relevant patents to highlight translational progress. Finally, current challenges, regulatory considerations, and future perspectives are discussed, underscoring the potential of nanoemulgels as a versatile and effective platform for advanced topical drug delivery.

## 1. Introduction

The fundamental aim of topical drug delivery is to maximize local therapeutic effects in the skin, such as in the treatment of inflammation, infections, pruritus, psoriasis, and dermatitis. These formulations are designed to deliver the therapeutic actives to act at or near the site of application rather than entering the general circulation, thereby resulting in lower overall exposure and a reduced risk of adverse effects [1]. Both topical and transdermal drug delivery systems face notable challenges, including a slow onset of action and the risk of skin irritation, which may vary depending on skin condition. A major limitation of cutaneous drug delivery is the skin’s intrinsic barrier function, which restricts penetration to protect the body from external insults, such as particulate matter, microorganisms, and drugs [2]. A major challenge in skin drug delivery is achieving adequate drug absorption, as only a limited proportion of the administered dose successfully penetrates the stratum corneum (SC) [3]. The effectiveness of skin drug delivery depends mainly on the nature of the drug molecule as well as on advanced design and formulation strategies that enhance permeation across the skin [4]. Since skin properties vary among individuals and across different body sites, and can be further altered by environmental exposure, and skin disorders, site-specific considerations are essential during formulation design to ensure effective drug delivery performance. For instance, age-related changes in skin, including reduced water content and lower enzymatic activity, significantly affect drug transport, leading to decreased permeation of hydrophilic molecules, altered prodrug activation, and increased deposition of nanoparticles [5]. Therefore, the successful design of skin-based delivery systems requires careful consideration of these variables to ensure optimal drug penetration and therapeutic effectiveness.

## 2. Skin Structure and Barriers

### 2.1. Skin Architecture and Its Implications for Cutaneous Drug Delivery

**Figure 1:**
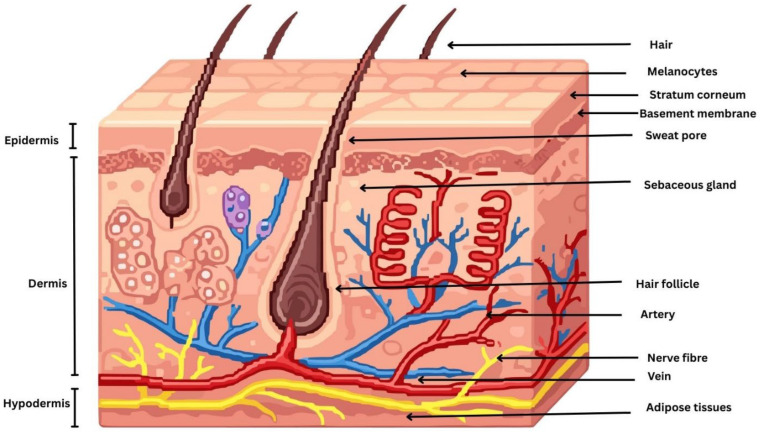
Schematic diagram illustrating the morphology of the skin.

The skin is a complex, multifunctional organ composed of three principal anatomical layers: the epidermis, the dermis, and the hypodermis. These layers are structurally and functionally distinct, working synergistically to provide a protective barrier, regulate thermoregulation, facilitate sensory perception, and support immunological and metabolic functions of the body [6]. Topical drug delivery primarily targets the epidermis and superficial dermis, where most localized skin disorders originate. The epidermis consists of the stratum basale, stratum spinosum, stratum granulosum, and SC, with drugs intended to act mainly within the viable epidermal layers. The stratum basale contains keratinocytes, melanocytes, and merkel cells, while the stratum spinosum and stratum granulosum are rich in differentiating keratinocytes and langerhans cells involved in immune regulation. The SC is a highly keratinized outer skin layer, approximately 10–20 µm thick and composed of 15–30 corneocyte layers organized in a characteristic brick-and-mortar architecture, which constitutes the primary barrier to topical drug delivery [7]. The epidermal basement membrane is a specialized interface that anchors the epidermis to the underlying dermis and plays a critical role in maintaining skin structural integrity and stability [8]. Disruption of this membrane can impair skin architecture, cellular metabolism, and regenerative capacity, leading to a range of skin disorders and, in severe cases, contributing to the development of serious dermatological diseases. Beneath the epidermis lies the dermis, a thicker and structurally complex layer composed predominantly of connective tissue rich in collagen and elastic fibers synthesized by sparsely distributed fibroblasts, which together provide mechanical strength and elasticity to the skin [9]. The dermis contains blood vessels, nerves, skin appendages, and specialized sensory cells that support skin function and sensory perception. Blood vessels are predominantly located in the dermis; however, under pathological conditions such as warts, nevi, and skin cancers, vascular proliferation and extension toward the epidermis may occur [10]. Below the dermis lies the hypodermis, or subcutaneous tissue, the deepest layer of the skin. Skin properties are influenced by environmental factors (e.g., UV radiation, pollutants, and toxins), lifestyle factors (e.g., diet and smoking), and physiological factors (e.g., aging, hormonal changes, and stress), which may disrupt skin homeostasis and contribute to dermatological disorders, including acne, skin melanoma, dermatitis, eczema, and psoriasis [11,12]. Understanding the structural and cellular composition of these layers is essential for designing effective topical drug delivery systems that achieve localized therapeutic action [13]. A schematic diagram illustrating the morphology of the skin is depicted in Figure 1.

### 2.2. Drug Transport Pathways Across the Skin

The SC forms a strong hydrophobic barrier that restricts the penetration of exogenous substances, including drugs and toxins [14]. This barrier function arises from flattened, keratin-rich corneocytes embedded in intercellular ceramide-based lipid bilayers that restrict molecular penetration. Modifying key stages of percutaneous absorption, particularly partitioning and diffusion, can enhance drug delivery to the skin [15]. Drug diffusion across this barrier occurs through a combination of lateral movement within lipid domains and trans-bilayer transport [16]. Computational modeling studies have demonstrated that diffusion within the SC lipid bilayers is predominantly lateral and occurs at rates significantly higher than perpendicular diffusion, thereby facilitating the transport of highly lipophilic molecules [17]. Effective percutaneous delivery requires that a drug reaches the skin surface in adequate amounts and at an appropriate rate. Permeation across the SC requires drugs to possess balanced solubility in both lipid and aqueous phases, a low molecular weight (typically <500 Da), and moderate lipophilicity (log P) ranging between 1 and 3 [18]. Among these factors, lipophilicity and molecular weight are the most influential determinants of skin permeability, as quantitatively described by the Potts and Guy equation, log P = −6.3 + 0.71 log Ko/w − 0.061 MW [19]. The dissolution and diffusion of drug molecules through the skin are significantly influenced by physicochemical properties such as melting point, pKa (dissociation constant), and overall charge [20].

Skin penetration in cutaneous drug delivery occurs primarily through three pathways: transcellular, paracellular (intercellular), and appendageal routes, with the extent of penetration governed by the physicochemical properties of both the drug and nanocarrier [21]. In the transcellular route, molecules pass directly through corneocytes, alternating between hydrophilic intracellular domains and lipophilic membrane regions. Because this pathway favors small, lipophilic, and uncharged molecules, permeation is strongly influenced by a drug’s partition coefficient, molecular weight, and solubility within SC lipids [22]. Although this pathway is the most direct, repeated transitions between aqueous and lipid phases limit its contribution to overall absorption, making it a minor route unless nanocarriers enhance membrane fluidity or improve drug solubility [23]. In the paracellular route, drug molecules diffuse through the tortuous lipid channels between densely packed keratinocytes, a process that avoids cellular damage and favors lipophilic compounds. However, the hydrophilic molecules face significant resistance due to the highly ordered lamellar lipid structure of the SC [24]. The transappendageal or follicular pathway contributes only ~0.1% of the total surface area, which involves drug penetration through hair follicles, sebaceous glands, and sweat ducts. It facilitates the entry of macromolecules, peptides, proteins, vaccines, and ionic compounds that poorly permeate the lipid-rich SC, with hair follicles acting as reservoirs for sustained delivery and sweat ducts providing hydrophilic pathways for charged molecules [25]. Nonetheless, its relevance is growing for nanoparticle-based delivery, vaccines, and targeted follicular therapy, where particle size, charge, and flexibility influence follicular uptake and retention. Although the appendageal route represents a small percentage of the skin surface, hair follicles and sebaceous glands serve as efficient low-resistance pathways, acting as reservoirs that retain nanoparticles and support sustained or targeted delivery, particularly for lipophilic drugs used in topical conditions [26].

Although the penetration of highly hydrophilic actives into the SC remains debated, evidence suggests the existence of an alternative corneocytary diffusion pathway involving corneocyte remnants and corneodesmosomes [27,28]. Small hydrophilic molecules (e.g., short peptides) can diffuse into the SC and corneocyte-associated regions, and their transport may be enhanced by hydrophilic diffusion enhancers such as N-methyl-2-pyrrolidone, 2-pyrrolidone, and Transcutol^®^. This pathway also offers potential for nano-based topical delivery systems, as appropriately engineered nanocarriers may modify local microenvironments to improve hydration and facilitate the delivery of larger hydrophilic biomolecules into the skin. Together, these mechanisms determine drug localization within the skin and are critical considerations in the design of effective topical formulations aimed at achieving localized therapeutic action.

## 3. Strategies of Skin-Based Drug Delivery

### 3.1. Transdermal and Topical Approaches

Transdermal and topical drug delivery systems both utilize the skin as a route of administration but differ in their therapeutic objectives and formulation design [29]. Transdermal drug delivery is intended to transport active pharmaceutical ingredients across the skin and into the systemic circulation to produce systemic effects and is commonly employed for drugs requiring sustained release, such as hormones (e.g., estradiol, testosterone), analgesics (e.g., fentanyl, buprenorphine), nicotine, antianginal agents (e.g., nitroglycerin), antihypertensives (e.g., clonidine), antiemetics (e.g., scopolamine), and antiparkinsonian agents such as rotigotine [30]. In contrast, topical drug delivery is primarily designed to exert localized therapeutic effects at or near the site of application, with minimal systemic absorption. This route is widely used for the treatment of dermatological conditions, including infections, inflammation, wound healing and skin disorders, and is optimized to enhance local drug retention rather than systemic pharmacokinetic control [31]. Topical drug delivery for skin diseases has traditionally relied on conventional formulations such as creams, ointments, lotions, gels, and pastes, which are effective for delivering drugs to superficial skin layers but often suffer from limitations including poor skin penetration, low drug stability, and variable therapeutic efficacy [32]. In recent years, nanotechnology-based delivery systems such as liposomes, niosomes, solid lipid nanoparticles, nanostructured lipid carriers, polymeric nanoparticles, and nanoemulsions have gained significant attention due to their ability to enhance skin penetration, improve drug solubility and stability, provide controlled drug release, and reduce local and systemic adverse effects [33,34,35].

### 3.2. Physiological and Physicochemical Determinants of Percutaneous Drug Absorption

Skin physiology plays a crucial role in percutaneous drug absorption, with variations in the thickness and lipid composition of the SC across different anatomical sites significantly influencing drug permeation [36]. Studies evaluating various chemically formulated gels and solutions demonstrated that percutaneous absorption varies by body region and is influenced by both the physicochemical properties of the formulation and the site of application [37]. Higher absorption was observed from the head, neck, and genital areas, while the trunk, back, and thighs showed comparatively lower uptake. Percutaneous absorption is enhanced in preterm neonates, infants, and young children due to better epidermal hydration, a thinner and more highly perfused SC, and a larger body surface area to body mass ratio, necessitating cautious use to avoid overdosage [38]. Ceramides are essential components of the SC lipid matrix and play a critical role in maintaining skin barrier integrity by limiting transepidermal water loss and restricting the penetration of external substances [39]. Different ceramide subclasses influence the skin barrier to varying extents; for example, ceramide NS is associated with barrier impairment, whereas ceramide NP is linked to intact and healthy skin. Molecular dynamics simulations show that NP-containing membranes have much lower water permeability than NS-containing membranes due to structural differences, confirming their role in skin permeability control. The density of dermal capillary blood vessels influences systemic drug uptake by maintaining a favorable concentration gradient, while hair follicles and sweat ducts provide alternative pathways that enhance drug penetration via the transfollicular route [40]. In addition, increased body temperature promotes vasodilation and elevated skin blood flow, resulting in higher absorption rates. A computational heat–mass transfer model showed that increasing skin temperature enhances transdermal drug absorption, with a 10 °C rise causing about a two-fold increase in nicotine uptake due to diffusion through the SC [41]. The model also demonstrated effective dermal clearance via capillaries and accurately predicted human pharmacokinetic data under normal and elevated temperatures. The use of occlusive systems enhances SC hydration, leading to increased dermal and transdermal drug absorption by swelling corneocytes, altering lipid organization, raising skin temperature, and increasing blood flow, making occlusive systems highly relevant in drug delivery [42].

A general classification system was developed to relate drug skin permeation and retention to key physicochemical properties and drug–lipid interactions, providing a theoretical basis for the rational design and evaluation of topical and transdermal drug delivery systems [43]. Topical formulations aim for high skin retention with minimal systemic absorption to reduce side effects, whereas transdermal systems and some topical products are designed to deliver drugs into the systemic circulation or subcutaneous tissues with lower skin retention to avoid local irritation [43]. However, drug permeation and skin retention occur simultaneously, and even locally applied drugs may enter systemic circulation [44]. Conventional topical formulations rely on passive diffusion and are suitable only for low molecular weight, moderately lipophilic, and highly potent drugs administered at low doses. Drug–vehicle interaction techniques such as prodrug selection, ion pairing, eutectic system formation, and thermodynamic enhancement represent second-generation transdermal strategies designed to enhance skin absorption without damaging deeper skin tissues [45]. The prodrug approach enhances skin absorption by increasing drug hydrophobicity through covalent modification, allowing better penetration of the SC and subsequent conversion to the active drug after absorption [46]. Ion pairing improves transdermal delivery of ionized drugs by forming neutral complexes that enhance SC permeation and dissociate after skin penetration to release the active drug [47]. Despite their advantages, drug–vehicle interaction methods have limitations, including potential toxicity from unpredictable prodrug metabolism, possible toxicity of linking agents or byproducts, complex and limited synthesis processes, and continued reliance on passive diffusion, with the SC remaining a major barrier to transdermal drug delivery [48].

**Figure 2:**
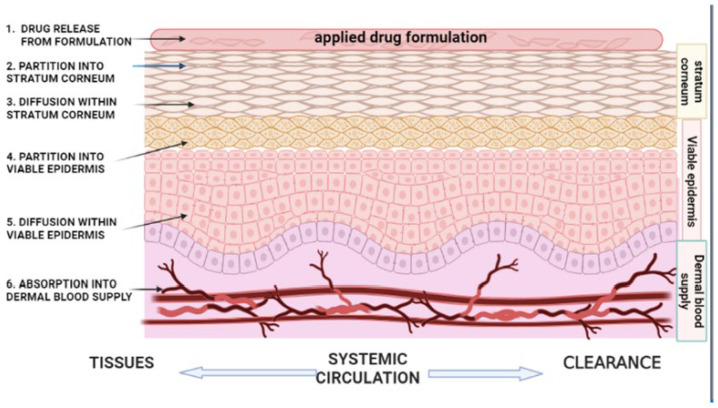
Schematic illustration of the kinetics of percutaneous drug absorption. The process involves: (1) release and partitioning of the drug from the applied topical formulation vehicle; (2,3) partitioning into and diffusion of the drug across the stratum corneum, which represents the rate-limiting step for most drugs; (4,5) partitioning into and diffusion through the viable epidermis, constituting the rate-limiting step for highly lipophilic drugs; and (6) uptake of the drug into the dermal microcirculation, followed by entry into the systemic circulation for distribution and clearance. This figure is adapted from [45], used under a CC BY 4.0 license.

Mathematical models based on Fick’s law of diffusion are commonly applied to quantify drug transport across the skin under steady-state conditions. The steady-state flux (*J*) of a drug is described by: J = dq/dt = DPCv/h where *q* is the cumulative amount of drug permeated per unit area over time (*t*); *D* denotes the diffusion coefficient within the skin; *P* is the partition coefficient between the drug, vehicle, and skin; C is the drug concentration in the vehicle; and *h* corresponds to the thickness of the skin barrier. This relationship highlights the critical influence of both physicochemical drug properties and skin barrier characteristics on topical and transdermal drug delivery performance [49]. The diffusion coefficient and effective skin thickness are physiologically determined parameters that critically affect the physicochemical processes underlying transdermal molecular transport [50]. Percutaneous absorption begins with the release of the drug from the formulation into the SC, a process largely governed by the drug’s thermodynamic activity, with higher activity levels leading to enhanced dermal penetration [51]. The efficacy of topical drug delivery is governed by the drug’s solubility in both the formulation vehicle and the lipid matrix of the SC, which controls its ability to overcome the SC barrier [52]. As the SC constitutes the primary rate-limiting barrier to skin permeation, drugs released from the vehicle must first partition into SC lipids and proteins before diffusing into the viable epidermis and dermis. This partitioning behavior is quantitatively described by the partition coefficient, defined as the ratio of drug concentration in the SC to that in the vehicle [53]. Drug–vehicle and skin–vehicle interactions critically influence drug release and skin permeation by affecting solute availability, release rate, partitioning behavior, and the diffusional resistance and hydration of the SC, thereby modulating overall transdermal flux [54]. An illustration explaining the sequential steps involved in percutaneous drug absorption is depicted in Figure 2.

## 4. Nanocarrier-Based Systems for Cutaneous Drug Delivery

### 4.1. Nanocarriers in Skin Delivery

Nanovesicles are nanosized lipid or surfactant-based bilayer vesicles, such as liposomes, ethosomes, transfersomes, transethosomes, niosomes, cubosomes, invasomes, and phytosomes, that enhance drug penetration through the skin by improving drug solubilization, modulating and fluidizing SC lipids, providing protection against drug degradation, and enabling controlled and targeted transdermal drug delivery. Nanovesicular systems enhance dermal and transdermal drug delivery through distinct yet complementary mechanisms [55]. Polymeric micelles, formed by the self-assembly of amphiphilic block copolymers, enhance dermal and transdermal drug delivery by solubilizing poorly water-soluble drugs within their hydrophobic core, improving formulation stability, and enabling controlled release, although their skin penetration is generally limited without the use of penetration-enhancement strategies [56]. Lipid–polymer hybrid nanoparticles combine the stability and controlled release of polymeric cores with the skin affinity of lipid shells, resulting in enhanced penetration and prolonged drug action [57,58]. Nanoemulsions enhance percutaneous drug delivery by improving drug solubility, increasing skin permeation, and facilitating controlled transport across the SC while maintaining skin barrier integrity [59,60]. A comparative evaluation of nanoemulsions and other nanocarrier systems used for cutaneous drug delivery is summarized in Table 1.

### 4.2. Mechanistic Insights into Nanocarrier-Mediated Cutaneous Drug Delivery

Nanocarrier-based cutaneous delivery systems enhance drug permeation by leveraging multiple physicochemical and biological mechanisms that help to overcome the formidable barrier of the SC. Their nanoscale particle size allows intimate contact with the skin surface, thereby improving adhesion and occlusion and facilitating passage through narrow intercellular channels. A recent study demonstrated that cell-penetrating peptide-decorated curcumin-loaded nanoemulsions with nanoscale droplet sizes (<100 nm) significantly enhanced skin penetration by facilitating transport through intercellular lipid pathways of the SC, while confocal laser scanning microscopy confirmed predominant drug retention within the epidermal layers and hair follicles in psoriatic skin, highlighting the superiority of optimized nanoemulsion systems over conventional formulations for cutaneous drug delivery [61]. Understanding how nanaocarrier properties influence skin penetration is crucial for improving dermal drug delivery, yet the role of particle size remains debated because it differs across nanoparticle types [62]. Reported optimal sizes for follicular penetration vary widely, for example, about 640 nm for PLGA particles [63], 80 nm for nanoemulsions [64], and 40–250 nm for polystyrene nanoparticles [65], showing that size effects are highly material-dependent. Mechanistic studies showed that particle size ~100 nm and the viscosity of the aqueous phase in oil-in-water emulsions strongly affect the penetration of poorly soluble, low-permeability ceramides into the epidermis and dermis [66].

Although surface charge is known to influence nanocarrier skin penetration, there is no clear agreement on which charge is most effective. The skin is generally considered negatively charged due to the abundance of anionic lipids in the SC [67]. Some studies report that neutral nanocarriers penetrate more efficiently, suggesting that positively charged particles may be trapped in superficial layers and negatively charged ones may be repelled by the skin barrier [68]. Other findings show that cationic nanomedicines exhibit strong electrostatic attraction to the negatively charged skin surface, enhancing retention and potentially improving treatment outcomes for dermatological disorders. A study investigating luteolin-loaded cationic nanoemulsions (CNEs) demonstrated that electrostatically charged nanocarriers significantly enhance transdermal delivery and skin retention [69]. The optimized formulation (CNE4), with a nanoscale droplet size (~112 nm) and positive zeta potential (+26 mV), showed superior stability, drug release, and ex vivo skin permeation compared with anionic nanoemulsions and drug suspension. Notably, cationic nanoemulsions achieved markedly higher permeation flux and drug deposition within skin layers, attributed to nanosization, surfactant-induced modulation of the SC lipid matrix, and electrostatic interactions with negatively charged skin components, highlighting their potential for improved cutaneous drug retention and transdermal therapy. Another study demonstrated that miconazole nitrate-loaded cationic nanoemulsion gels provided significantly higher skin permeation and dermal drug retention than anionic systems across artificial membranes, EpiDerm, and rat skin [70]. The enhanced performance of cationic formulations was attributed to electrostatic interactions with skin components, optimized formulation composition, and increased skin hydration, with imaging studies confirming deeper penetration, supporting their potential for treating deep-seated fungal infections. Conversely, negatively charged clove oil nanoemulsions demonstrated prolonged antibacterial activity against Gram-positive bacteria, including *Listeria monocytogenes* and *Staphylococcus aureus* [71]. Despite the absence of electrostatic attraction, the anionic droplets appeared to self-assemble with bacterial membranes and interact with intracellular components, contributing to their sustained antimicrobial effect.

Deformability of nanoparticles can be enhanced by incorporating membrane-softening agents like deoxycholic acid, allowing carriers to better navigate complex biological environments and improve drug delivery efficiency. Increased deformability is particularly valuable for enhancing skin penetration without disrupting the SC barrier [72]. System deformability, particularly in ultraflexible vesicles such as transfersomes and ethosomes, enables particles to squeeze through constricted intercellular spaces under hydration gradients or osmotic forces, significantly enhancing penetration. Ionic liquids enhance deeper skin penetration by fluidizing SC lipids, improving drug solubility and partitioning into skin layers, increasing nanoemulsion droplet flexibility, and boosting skin hydration. These combined effects open intercellular pathways and facilitate more efficient transport of drugs into deeper epidermal regions. A potent dacarbazine derivative, HIT-1, showed strong anti-melanoma activity, and an oil-in-oil ionic liquid nanoemulsion was developed using the highly permeable, biocompatible L-pyrrolidone carboxylic acid-matrine ionic liquid (P-M IL) to enhance delivery [73]. Among the formulations, HIT-1/PM-ME-1-1 achieved the best skin penetration and antitumor effects, effectively activating apoptosis pathways and stimulating immune responses. Nanoemulsions have been shown to interact with the SC lipid matrix, effectively fluidizing intercellular lipids and reducing barrier resistance, which helps to create alternative permeation pathways and facilitates deeper drug penetration into the skin layer [74].

Due to their small particle size, nanocarriers adhere strongly to the SC, forming an occlusive layer that enhances skin hydration, loosens corneocyte packing, widens intercellular spaces, and consequently improves permeant penetration across the skin barrier [75]. Additionally, studies reported that nanoemulsions create an occlusive effect due to the fluid lipids in their matrix [76].

Many nanocarriers, such as microemulsion and nanoemulsion, incorporate surfactants or lipid components that transiently disrupt or fluidize the SC lipid matrix, reducing its packing density and thereby lowering the diffusional resistance [77]. Furthermore, many nanocarriers enhance drug solubility and thermodynamic activity, creating a strong concentration gradient that drives transdermal flux. In some systems, such as flexible vesicles, nanoparticles, and lipid nanosystems such as nanoemulsion, they have the ability to localize within hair follicles which provides an additional reservoir for sustained release and deeper follicular delivery. It was reported that clove oil-based minoxidil nanoemulsions enhanced follicular drug penetration by more than 26-fold compared with control formulations, highlighting their strong potential for targeted topical treatment of alopecia [78]. Collectively, these mechanisms including size-dependent permeation, SC disruption, hydration enhancement, charge-mediated interactions, and structural deformability enable nanocarrier systems to markedly improve dermal penetration and systemic absorption compared with conventional topical formulations.

### 4.3. Nanoemulsions as Versatile Platforms for Dermal Drug Delivery

Nanoemulsions are increasingly recognized as superior carriers for dermal and transdermal drug delivery due to their nanosized droplet structure, which provides a large surface area, improved solubilization of poorly water-soluble drugs, and enhanced dispersion within the skin [79]. The dermal and transdermal efficacy of nanoemulsion-based systems has also been demonstrated for water-soluble drugs, such as levamisole [80]. Their small droplet size and surfactant components facilitate greater drug partitioning into and permeation across the SC, overcoming its barrier limitations more effectively than many other nanocarriers [81]. In addition, nanoemulsions can be formulated with stable kinetic properties and flexible composition, allowing both localized dermal retention and systemic transdermal uptake depending on the therapeutic goal [81,82]. Compared with rigid nanoparticles or vesicular systems, their formulation simplicity, physical stability, and ability to incorporate a wide range of hydrophilic as well as hydrophobic actives make them particularly advantageous for both research and clinical translation in skin delivery applications. Nanoemulsions are well recognized in topical drug delivery due to their ability to form uniform films on the skin and effectively overcome the SC barrier, thereby enhancing dermal penetration and drug retention [83]. Numerous in vivo and in vitro studies have demonstrated the feasibility and effectiveness of micro and nanoemulsions for skin drug delivery, supporting their application as lipid-based topical formulations [84,85,86]. In addition, nanoemulsions may be integrated into other nanoscale delivery platforms, such as vesicular systems or lipid carriers, where they can function as internal or external phases to enhance drug loading, stability, and skin permeation. Phonophoresis uses ultrasound to enhance transdermal drug absorption by disrupting the SC, and a 2020 clinical study demonstrated that phonophoresis-assisted nanoemulsion delivery significantly improved drug permeation, safety, and therapeutic outcomes in knee chondropathies [87]. Microneedling is a minimally invasive technique that enhances cutaneous delivery by creating microscopic channels in the SC using fine needle arrays or rollers, typically without significant pain or bleeding. Studies have demonstrated that pretreatment of the skin with microneedles significantly improves the transdermal delivery of nanoemulsion-based systems, such as MF59-adjuvanted influenza formulations, by increasing antigen penetration into deeper skin layers [88].

### 4.4. Nanoemulgels for Topical Drug Delivery

Despite offering several advantages, including favourable physicochemical properties and improved drug thermodynamic stability, nanoemulsions suffer from inherently low viscosity, which limits their spreadability, bioadhesion, and residence time on the skin surface [89]. This fluid nature often results in rapid runoff, nonuniform film formation, and insufficient contact with the SC, thereby reducing drug absorption efficiency and contributing to dose variability, which collectively hinder their clinical translation for topical and transdermal applications. To overcome these limitations, nanoemulsions are incorporated into three-dimensional hydrogel networks to form nanoemulgels, thereby improving viscosity, spreadability, adhesiveness, residence time on the skin, and patient acceptability while retaining the penetration-enhancing properties. As topical or transdermal administration systems, they function as drug reservoirs, facilitating the controlled release of the drug from the dispersed phase to the external or continuous phase, and subsequently onto the skin [90]. Typically formulated using aqueous or hydroalcoholic bases and polymeric gelling agents such as carbomers, cellulose derivatives, or poloxamers, nanoemulgels entrap nanoemulsion droplets within a hydrated polymer matrix, thereby increasing viscosity, improving structural integrity, promoting sustained drug release, improved local drug concentration at the application site thereby resulting in overall performance [91,92]. From a physicochemical standpoint, this structural integration enhances kinetic stability by restricting droplet mobility and Brownian motion, which reduces the frequency of droplet collisions and mitigates destabilization processes such as coalescence, flocculation, and creaming, while polymer–surfactant interactions at the oil–water interface may further strengthen interfacial films through steric stabilization [93]. In addition, polymer–surfactant interactions can also change microstructure and drug partitioning, so polymer and surfactant levels should be optimized for each drug [94]. Although molecular diffusion-driven phenomena such as Ostwald ripening may still occur, the gel matrix significantly retards droplet growth by limiting macroscopic movement of the dispersed phase. Moreover, the hydrogel network plays a critical role in modulating drug release kinetics, as drug transport from nanoemulgels typically follows a multistep mechanism involving partitioning from the internal oil phase, diffusion through the polymeric gel matrix, and subsequent permeation across the skin barrier. This multilevel barrier often results in sustained or controlled drug release, enhanced skin retention, and prolonged local drug concentrations, making nanoemulgels particularly advantageous for dermal and transdermal delivery of drugs with short biological half-lives, frequent dosing requirements, or narrow therapeutic windows [95]. Importantly, increasing viscosity can enhance residence time, but excessive gel strength may reduce spreadability and hinder drug diffusion/permeation; hence, an optimal balance between retention and release/permeation is required for consistent therapeutic performance [96]. Accordingly, nanoemulgel development should be supported by practical performance testing, evaluation of skin tolerability and irritancy. In summary, nanoemulsion-based hydrogels effectively overcome limitations of conventional drug formulations and have gained increasing research interest due to their ease of application, good spreadability, non-sticky nature, safety, and therapeutic effectiveness.

## 5. Components of Nanoemulsion

The composition and maximum allowable concentrations of FDA-approved excipients used in nanoemulsion formulations are determined by the intended route and purpose of administration. The development of nanoemulsions is therefore restricted to components that are considered safe and generally recognized as safe (GRAS), including oils, surfactants, emulsifying agents, polymers, viscosity and density modifiers, and ripening inhibitors. Current evidence suggests that systemic exposure to most nanoemulgel excipients after topical use is typically low because the SC restricts the penetration of many surfactants and, especially, large hydrophilic polymers [97]. Since human pharmacokinetic data for nanoemulgel excipients after skin penetration are limited, regulators rely on conservative exposure assumptions, excipient history of use (e.g., Inactive Ingredients Database), irritation/sensitization testing, and, when needed, systemic safety assessments based on estimated exposure.

### 5.1. Oil Phase

The oil phase represents a critical component, as it serves as the primary solubilizing medium for lipophilic drugs and exerts a decisive influence on droplet size, drug loading capacity, release kinetics, and overall formulation stability [98]. The selection of an appropriate oil phase is guided by drug solubility, the required hydrophilic–lipophilic balance (HLB), the intended site of action, and safety considerations [99]. In dermal and transdermal systems, the oil phase also plays a key role in modulating skin permeation by interacting with SC lipids; certain oils act as penetration enhancers by disrupting the highly ordered lipid domains of the skin barrier, thereby facilitating drug transport [100]. For dermal applications, oils that favour localized drug retention with minimal systemic absorption are generally preferred, whereas transdermal formulations typically employ oils with strong permeation-enhancing capabilities [101]. When natural oils are used, HLB values greater than 10 generally favor the formation of oil-in-water nanoemulsions, while values below 10 tend to result in water-in-oil systems. The selection of an appropriate oil phase requires balancing its drug-solubilizing capacity with its ability to form a stable nanoemulsion system [102].

Typically, oil-in-water nanoemulsions contain 5–20% dispersed oil phase, although lipid concentrations as high as 70% have been reported in certain systems [103]. Due to their optical and mechanical characteristics, lipid-based interfacial films may be relatively thick, brittle, and translucent. Re-esterified fractions obtained from natural fixed oils such as coconut, sesame, sunflower, and cottonseed oils categorized as long, medium, and short-chain triglycerides are widely employed as oil phases to optimize solubilization and stability in nanoemulsion systems [104]. In addition, a wide range of synthetic lipids such as Capryol^®^ 90, triacetin, isopropyl myristate, palm oil esters, isopropyl palmitate, Labrafil M1944CS, Maisine 35-1, Miglyol^®^ 812, and Captex derivatives are frequently utilized in nanoemulsion formulations due to their favorable solubilization and formulation properties [105]. These lipid-based excipients are commonly classified as oil-phase components or lipophilic co-surfactants rather than conventional surfactants. Owing to their low HLB values and amphiphilic nature, these excipients enhance drug solubilization, facilitate self-emulsification, and contribute to nanoemulsion stability while also acting as penetration enhancers in dermal and transdermal formulations. Natural oils are widely used as oil-phase components in nanoemulsion formulations due to their excellent biocompatibility, ability to solubilize lipophilic drugs, and function as natural penetration enhancers. For example, cinnamon oil has been employed as the oil phase to dissolve tadalafil in a transdermal nanoemulgel developed for the treatment of Raynaud’s phenomenon, demonstrating effective transdermal delivery and offering a patient-friendly alternative to oral therapy [81]. Many essential oils also possess intrinsic antimicrobial and antioxidant properties that can improve formulation stability and therapeutic performance [106]. Nanoemulsions formulated with oils of very low aqueous solubility, and with optimized interfacial compositions, exhibit suppressed Ostwald ripening and improved kinetic stability, highlighting the importance of both interfacial properties and phase solubility in maintaining droplet uniformity over time. This interplay between interfacial flexibility and phase solubility is a key focus in contemporary nanoemulsion research aimed at enhancing long-term stability across pharmaceutical, food, and cosmetic applications [107]. In summary, oil selection must balance drug solubilization with the intended dermal or transdermal effect, since highly permeation-enhancing oils may also increase irritation and reduce local retention [108]. Oils with higher aqueous solubility can promote Ostwald ripening, so long-term stability requires low-solubility oils and optimized interfacial composition. The key oil-phase components used in dermal and transdermal nanoemulsion formulations are depicted in Table 2.

### 5.2. Emulsifying Agents

The primary function of emulsifying agents is to facilitate nanoemulsion formation through multiple mechanisms, including reduction in interfacial tension, formation of a rigid interfacial film that acts as a mechanical barrier, and development of an electrical double layer that prevents droplet–droplet interactions. These mechanisms collectively maintain kinetic stability by inhibiting flocculation, creaming, coalescence, and phase separation [105]. The interfacial layer between the oil and water phases is critical for emulsion stability, as its physicochemical properties largely determine the behaviour of dispersed droplets. Characteristics such as interfacial layer thickness, structure, interactions among adsorbed emulsifiers, and interfacial rheological properties play a key role in the formation and stabilization of emulsions [109]. The polysaccharide emulsifier (e.g., pectin, alginate, xanthan, octenyl succinic anhydride) forms a thick interfacial layer that stabilizes the emulsion primarily through spatial repulsive forces. In contrast, globular proteins (e.g., soya and whey protein isolate, ovalbumin, bovine serum albumin) create thinner interfacial layers that stabilize emulsion droplets via a combination of electrostatic interactions and steric repulsion. Electrostatic stabilization occurs when emulsifying agents impart surface charges to dispersed droplets, creating repulsive forces that prevent coalescence, while steric stabilization is achieved when non-ionic surfactants or polymers form a protective, hydrated layer around droplets that acts as a physical barrier [110]. Electrostatic stabilization is sensitive to pH and electrolyte concentration, whereas steric stabilization is more robust under varying physiological conditions. In some formulations, emulsifying agents provide electrosteric stabilization by combining both mechanisms, resulting in enhanced emulsion stability. Phospholipids, commonly used as emulsifiers in the form of lecithin, are amphiphilic molecules that stabilize emulsions by adsorbing at the oil–water interface and reducing interfacial tension through interfacial film formation and electrostatic repulsion [111]. The selection of an appropriate emulsifier and its concentration is critical and should consider both the HLB and critical packing parameter, as these factors govern the formation of a stable, coherent, and flexible interfacial film that effectively prevents droplet coalescence [112]. Moreover, optimization of critical process parameters such as mixing conditions, order of addition, and temperature guided by Quality by Design principles, is essential to ensure consistent product quality and performance [113]. Based on the nature of the interfacial film formed, emulsifying agents may be classified as surface-active agents that form monomolecular films, hydrocolloid-based emulsifiers that produce multimolecular films, or finely divided solids that stabilize interfaces through particulate films. Proteins have been shown to act as effective emulsifiers in oil-in-water nanoemulsions by providing combined steric hindrance and electrostatic repulsion; however, their stabilizing efficiency is highly sensitive to pH and ionic strength, particularly near their isoelectric point, where aggregation may occur [113]. In contrast, polysaccharides are generally less suitable for nanoemulsion stabilization due to their high-water solubility and moisture absorption, which result in poor barrier properties in liquid formulations [114]. Emulsifier performance is strongly influenced by conditions such as pH, ionic strength, temperature, and dilution, which can reduce interfacial stability in real-use settings [115]. Although higher surfactant levels may improve stability, they can increase irritation risk, so emulsifier choice must balance stability with safety and scalability [116].

### 5.3. Surfactant/Cosurfactant

Surfactants and co-surfactants play a pivotal role in the formation and stabilization of nanoemulsions by markedly reducing interfacial tension between the immiscible oil and aqueous phases and facilitating the generation of nanosized droplets. The flexibility and resilience of the interfacial film formed by surfactant molecules are critical for the kinetic stability of nanoemulsions, as the interfacial film can rapidly reorganize following mechanical disturbance, maintain coverage of nascent oil–water interfaces, and reduce interfacial tension gradients that drive droplet coalescence [117]. Surfactants adsorb at the oil–water interface to form a dynamic interfacial film, while co-surfactants penetrate and fluidize this film, increasing its flexibility and enabling spontaneous curvature necessary for nanoemulsion formation. Short and medium-chain alcohols such as ethanol, butanol, pentanol, isopropanol, and propylene glycol were used as cosurfactants to lower interfacial tension and enhance interfacial fluidity, while also modifying the solubility of both the aqueous and oil phases through phase partitioning [118]. For instance, co-surfactants, typically short-chain alcohols or glycols, penetrate the surfactant monolayer, reduce packing constraints, and increase interfacial fluidity and elasticity, thereby facilitating spontaneous emulsification and minimizing droplet coalescence [119]. Other cosolvents/cosurfactants included polyethylene glycol (PEG) 400 and Transcutol^®^ HP. The synergistic action of surfactant and co-surfactant also expands the nanoemulsion existence region in pseudo-ternary phase diagrams and enhances the solubilization of lipophilic drugs within the interfacial region. Additionally, the presence of a co-surfactant allows for a reduction in the total surfactant concentration, thereby improving biocompatibility and reducing irritation potential. Collectively, these effects contribute to improved kinetic stability, enhanced drug loading capacity, and overall performance of nanoemulsions across different delivery systems, including dermal and transdermal routes. The preparation of nanoemulsions is significantly influenced by the selection and concentration of an appropriate surfactant, which facilitates rapid adsorption at the oil–water interface and stabilizes the newly formed nanoscale droplets that are subjected to high Laplace pressure often in the range of 10–100 atmosphere [120]. In addition to these interfacial effects, Ostwald ripening remains a dominant destabilization mechanism in nanoemulsions, driven by molecular diffusion of oil from smaller to larger droplets due to differences in chemical potential at curved interfaces.

Depending on their HLB, non-ionic surfactants such as polysorbates (Tween^®^ 20, 80), sorbitan esters (Span^®^ series), Lauroglycol^®^90, Cremophor^®^EL, or Cremophor^®^RH 40 are most commonly employed due to their greater physicochemical stability, safety, formulation flexibility, low toxicity, relative insensitivity to pH and ionic strength, superior emulsification efficiency, steric stabilization, and biocompatibility [59,121,122]. Polyoxyethylene sorbitan esters (Tween 20, 40, 60, and 80) are commonly used to stabilize oil-in-water (o/w) nanoemulsions, owing to their high HLB values. Combining high-HLB (e.g., Tween 80) and low-HLB (e.g., Span 20) non-ionic surfactants has been shown to improve interfacial film flexibility, resulting in smaller droplet size, enhanced stability, and controlled drug release through the SC. Non-ionic surfactants are increasingly paired with oils such as medium-chain triglycerides, isopropyl myristate, or essential oils, enhancing skin permeation while maintaining formulation mildness [123]. A recent pharmaceutics review notes that non-ionic surfactants dominate many nanoemulsion/microemulsion studies, largely due to pH insensitivity and established safety and it frames current formulation practice around commonly accepted non-ionic systems and ternary diagram approaches [124]. In contrast, sorbitan esters (Span 20, 40, 60, and 80), with lower HLB values, are frequently utilized as co-surfactants in water-in-oil (w/o) systems. Polyoxyethylene ether surfactants such as Brij^®^ 97 provide strong steric stabilization through hydrated PEG chains, enhancing nanoemulsion stability [125]. Polyglycerol esters of fatty acids exhibit high emulsification efficiency and improved tolerance to environmental stress conditions, while sugar-based surfactants such as sucrose monopalmitate are favored for their excellent safety profile, biodegradability, and regulatory acceptance. As reported in the literature, the irritation potential of surfactants is closely related to their aggregation behavior [126]. Highly irritating surfactants such as cocamidopropyl betaine and sodium alkyl benzene sulfonate form smaller and less stable aggregates, whereas moderately irritating surfactants such as lauroyl glucoside and sodium lauroyl sarcosinate form larger, more stable self-assembled structures in aqueous solutions.

Conversely, anionic surfactants (e.g., sodium lauryl sulfate, sodium dodecyl sulfate) and cationic surfactants (e.g., β-lactoglobulin) primarily stabilize nanoemulsion droplets through electrostatic repulsive forces, thereby reducing droplet aggregation. However, their performance is highly dependent on pH and ionic strength, and charge screening in the presence of electrolytes may compromise emulsion stability. Moreover, ionic surfactants are associated with membrane disruption, protein denaturation, and mucosal irritation at higher concentrations, which limits their applicability in formulations requiring elevated surfactant levels, particularly for parenteral, ocular, and mucosal delivery systems. Zwitterionic surfactants such as lecithin exhibit high interfacial activity and form compact interfacial films due to the presence of both positive and negative charges within the same molecule. These surfactants offer advantages including biodegradability, foam stability, low critical micelle concentration, high water solubility, and reduced irritation and toxicity compared to ionic surfactants. Nevertheless, their widespread pharmaceutical use is often constrained by higher production costs and variability in composition compared to synthetic non-ionic surfactants [127]. It is worth noting that even if small amounts of synthetic surfactants (e.g., Poloxamers) reach the viable epidermis/dermis and enter systemic circulation, these excipient classes are widely used and well evaluated in safety assessments. Any systemically absorbed PEG-like fractions are generally expected to be cleared mainly via renal excretion, with clearance influenced by molecular weight [128].

A liquid crystal nanoemulsion is a novel emulsion system in which emulsifier molecules self-assemble at the oil–water interface into a lamellar liquid crystalline structure resembling the lipid organization of the SC. Such nanoemulsions can be prepared by microfluidization of liquid crystal emulsions stabilized with hydrogenated lecithin and phytosterols, while retaining their ordered lamellar interfacial structure [129]. A recent study suggest replacing/combining conventional ethoxylated non-ionics with sugar-based surfactants such as alkyl polyglucosides and related glucosides (e.g., coco-glucoside) to improve biodegradability and skin/ocular tolerability while maintaining emulsification performance [130]. Another work highlights polyglycerol-based nonionic surfactants used with microfluidization, where formulation components (e.g., glycerol) and surfactant architecture are tuned to achieve uniform, small droplet sizes and improved stability [131].

Surfactant/cosurfactant selection improves droplet size and stability but may increase irritation or membrane disruption, particularly with alcohol cosurfactants and ionic surfactants [132]. Electrostatic stabilization can fail in electrolyte-rich or diluted conditions, and Ostwald ripening may still compromise long-term stability unless oil solubility and interfacial composition are optimized.

### 5.4. Auxiliary Agents

In addition to the fundamental components, pharmaceutical nanoemulsions often include auxiliary excipients to improve formulation performance and stability [133]. For example, cosolvents such as propylene glycol or PEG derivatives are used to enhance drug solubility and reduce interfacial tension, while viscosity-modifying polymers, including polysaccharides and proteins, are commonly incorporated into nanoemulsions to enhance stability and improve textural properties [134]. In nanoemulsions containing a lower-density oil phase, density modifiers or ripening inhibitors, such as sucrose acetate isobutyrate, are added to prevent creaming [135]. Additionally, incorporation of highly hydrophobic long-chain triglycerides into the dispersed phase reduces oil solubility in the aqueous phase and suppresses Ostwald ripening, which is particularly important in oil-in-water nanoemulsions formulated with slightly water-soluble oils such as essential oils and flavor compounds [104]. Furthermore, preservatives and antioxidants are incorporated to inhibit microbial growth and oxidative degradation, respectively, and buffers or pH modifiers ensure physiological compatibility and maintain stability during storage and administration [136,137]. In specialized systems, solubilizers (soluplus^®^) and penetration enhancers/cosolvents (carbitol^®^, dimethylacetamide), targeting ligands (e.g., hyaluronic acid, folic acid, ceramides, and arginine–glycine–aspartic acid peptides), or cryoprotectants (e.g., trehalose, mannitol, and polyvinylpyrrolidone) may be included to optimize bioavailability, site-specific delivery, or stability during drying processes [138]. These additional components are critical in tailoring nanoemulsion formulations for diverse therapeutic applications and improving their physicochemical and biological performance. Auxiliary excipients can improve nanoemulsion stability and performance, but they also add formulation complexity and may introduce compatibility, safety, and regulatory challenges.

### 5.5. Gelling Agents

Gelling agents play a crucial role in the conversion of nanoemulsions into nanoemulgels by imparting appropriate viscosity, structural stability, and semisolid consistency required for topical application [139]. These polymers form a three-dimensional network within the continuous phase, entrapping nanoemulsion droplets without compromising their nanoscale characteristics. The incorporation of a suitable gelling agent enhances formulation spreadability, improves physical stability by reducing droplet mobility, and prolongs residence time at the site of application. In addition, certain gelling agents exhibit bioadhesive and permeation-enhancing properties, facilitating prolonged drug–skin contact and improving dermal penetration. The selection of the gelling agent and its concentration significantly influences the rheological behavior, drug release profile, and overall therapeutic performance of nanoemulgels. Carbopol polymers (e.g., Carbopol 940, 934, and Ultrez^®^ 21), typically used at low concentrations (0.1–1.5%), are the most commonly employed gelling agents due to their high thickening efficiency, favorable rheological properties such as spreadability, and excellent compatibility with nanoemulsion systems [140]. Due to their high molecular weight, gelling polymers such as Carbopol^®^ have minimal percutaneous absorption, so they primarily act locally as viscosity modifiers rather than as systemically absorbed or metabolized components [141]. Their widespread use as inactive ingredients in approved products also indicates strong regulatory familiarity when used within acceptable concentration ranges. Cellulose derivatives such as carboxymethyl cellulose (3–6%) and hydroxypropyl methylcellulose (2–6%) are also widely utilized owing to their biocompatibility, neutral, resistant to microbial growth, non-irritant nature, and ability to impart desirable pseudoplastic flow behavior. In addition, Pluronic^®^ F127 (20–30%) and chitosan have been explored as alternative gelling matrices, offering thermoreversible gelation, bioadhesive characteristics, and, in some cases, enhanced drug permeation [142]. Collectively, these gelling agents enhance formulation viscosity, physical stability, spreadability, and skin retention, thereby improving topical drug delivery and therapeutic efficacy. Gelling agents enhance stability and skin residence time, but overly high polymer levels can reduce spreadability and drug diffusion, so the viscosity–permeation balance must be optimized [143]. Polymer choice also affects compatibility, pH/ionic sensitivity, microbial risk, and long-term rheological stability, which should be verified under realistic storage and use conditions.

## 6. Preparation Methods

Nanoemulsions for topical drug delivery are prepared using high-energy or low-energy emulsification methods.

### 6.1. High Energy Methods

High-energy methods are widely used for nanoemulsion preparation, employing intense mechanical energy to overcome interfacial tension between the oil and aqueous phases and reduce coarse emulsion droplets into stable, uniformly dispersed nano-sized droplets typically ranging between 20 and 200 nm. Common high-energy techniques include high-pressure homogenization (HPH), microfluidization, and ultrasonication.

#### 6.1.1. High Pressure Homogenization

In HPH, the coarse emulsion is forced through a narrow valve under very high pressure, generating shear stress, turbulence, and cavitation that break droplets into nanoscale sizes. For instance, HPH effectively produced stable limonene nanoemulsions by reducing droplet size to the nanoscale through intense shear, cavitation, and droplet collisions, outperforming other emulsification methods and maintaining stability for 28 days at room temperature [144]. A commercial high-pressure valve homogenizer with controllable pressure, nozzle geometry, flow pattern, and back pressure was systematically assessed to identify key operating parameters influencing nanoemulsion formation [145]. The results showed that higher homogenization pressure, multiple passes, increased back pressure, and higher emulsifier-to-oil ratios significantly reduced droplet size, with reverse flow configurations yielding slightly smaller droplets than parallel flow. The technique’s performance also depended on emulsifier type, with plant, animal, and synthetic emulsifiers exhibiting different size reduction efficiencies. Droplet size generally decreases as homogenization pressure, number of passes, back pressure, and the emulsifier-to-oil ratio increase, although the outcome also depends on the flow configuration and the emulsifier type [145].

#### 6.1.2. Ultrasonic Homogenization

It employs acoustic cavitation, where the collapse of microbubbles produces intense shear forces leading to droplet disruption. A study reported that ultrasonic emulsification is an efficient nanoemulsion technique for preparing phase change material nanoemulsion, where the formulation was optimized by controlling key process variables, namely ultrasonic amplitude, treatment time, and surfactant concentration [146]. Among these, surfactant concentration had the greatest influence, followed by ultrasonic amplitude and treatment time, on droplet size and viscosity. At optimized process conditions, ultrasonication produced stable nanoemulsion with droplet sizes around 118 nm, outperforming rotor–stator homogenization and phase inversion temperature (PIT) methods in terms of emulsion stability and lower viscosity, thereby demonstrating its suitability for high-performance nanoemulsion production. A stable water-in-oil nanoemulsion containing a phenolic-rich olive cake extract was prepared using two nanoemulsion techniques, ultrasonic homogenization and rotor–stator mixing and optimized using response surface methodology [147]. Ultrasonic homogenization at 20% amplitude for 15 min produced nanoemulsion with smaller droplet size (~105 nm) and lower polydispersity index (PDI), indicating superior droplet uniformity compared with rotor–stator mixing, which required high shear (20,000 rpm for ~10 min) to achieve a comparable size range.

An in-depth investigation examined the ultrasonication technique and proposed the existence of a constant optimal ultrasonication time that is largely independent of processing conditions [148]. Using oil-in-water nanoemulsion as a model system, the results showed that product parameters (oil and surfactant composition) significantly influenced droplet size and stability, whereas ultrasonication time, beyond a certain point, did not further reduce droplet size. An optimal ultrasonication time of ~10 min was identified and found to be consistent across different amplitudes, volumes, and oil systems. Ultrasonication was effectively used to prepare eucalyptus oil nanoemulsions by optimizing key process parameters, including sonication distance, amplitude, and time [149]. Under optimal ultrasonic conditions, nanoemulsion with a mean droplet size of ~19 nm, narrow size distribution, and high zeta potential were obtained. Ultrasonication significantly enhanced emulsion stability and antimicrobial activity compared to native eucalyptus oil, demonstrating its suitability as a simple and efficient method for nanoemulsion preparation. A recent investigation compared microfluidization and ultrasonication as nanoemulsion techniques by varying key process parameters [150]. Ultrasonication, optimized through amplitude and sonication time, produced smaller droplet sizes and showed superior emulsifying performance, protein adsorption, and thermal and centrifugal stability compared with microfluidization. In contrast, microfluidization, even at higher pressures and multiple cycles, resulted in larger droplets, indicating that ultrasonication was the more effective technique for generating stable, fine emulsions. Many studies suggest an optimal sonication time beyond which further processing gives little additional size reduction, emphasizing process and formulation optimization for reproducibility [148].

#### 6.1.3. Rotor–Stator Emulsification (RSE)

RSE is also referred to as high-speed homogenization or colloid mill variants, employs rapid rotation of a rotor within a stationary stator to create strong shear forces, enabling efficient droplet disruption during the pre-emulsification stage. A study reported that rotor–stator homogenization can be used as an effective alternative to HPH for the production of food-grade nanoemulsions [151]. The study highlighted that, unlike HPH, which are energy and maintenance-intensive and mainly suitable for dilute, low-viscosity systems, RSE can produce dilute to concentrated nanoemulsions with droplet sizes in the range of 100–500 nm. Moreover, it was demonstrated that modified starch produced stable nanoemulsions due to rapid interfacial adsorption, while gum arabic led to larger droplets because of drop–drop coalescence during emulsification. Investigation revealed that hydrodynamic cavitation employing a rotor–stator reactor is an efficient technique for the continuous production of oil-in-water nanoemulsions for skincare applications [152]. The integration of a 3D-printed rotor and optimization of key process and formulation variables including rotor speed, flow rate, surfactant concentrations, and oil content enabled the generation of submicron nanoemulsions (~366 nm). RSE is scalable and practical but often produces larger, less uniform droplets than HPH, sometimes requiring additional processing [153]. High shear may also promote coalescence and thermal/oxidative stress, so emulsifier choice and operating conditions must be optimized for stable nanoemulsions.

#### 6.1.4. High-Pressure Microfluidic Homogenization (Microfluidization)

Microfluidization technique reduces droplet size by forcing the coarse pre-emulsion through microchannels (interaction chambers), where controlled impact, intense shear, and turbulence produce nanoemulsions with narrow size distribution. Microfluidization has been widely employed as an efficient high-energy technique for the preparation of nanoemulsions containing poorly water-soluble bioactive compounds [154]. In this context, andrographolide-loaded nanoemulsions were produced by optimizing homogenization pressure and the number of processing cycles, which enabled control over droplet size, PDI, and surface charge. Optimal nanoemulsions were obtained at 20,000 psi with five cycles, demonstrating that microfluidization is a robust and scalable technique for generating stable, uniform nanoemulsions suitable for topical delivery systems.

#### 6.1.5. Hybrid or Combined High Energy Techniques

An integrated HPH system combining piston-gap and microfluidic technologies demonstrated its effectiveness as an advanced non-thermal nanoemulsification technique for lemon emulsions [155]. Application of this technique at 200–400 MPa reduced droplet size to the sub-500 nm range, enhanced emulsifying efficiency, and produced physically stable nanoemulsions with improved electrostatic stability. In addition, combined high-energy sequences such as ultrasonication followed by HPH, or high-speed mixing prior to HPH are increasingly used to improve process efficiency, reduce the number of homogenization cycles, and achieve superior droplet size uniformity. Comparative evaluation of ultrasound-assisted emulsification (UAE), HPH, and high-speed homogenization showed that UAE and HPH are more effective techniques for producing high-quality protein-stabilized oil-in-water emulsions than high-speed homogenization [156]. Among them, UAE emerged as the superior technique, yielding the smallest droplet size, highest interfacial protein adsorption, and best storage stability, indicating its strong potential for fabricating fine and stable nanoemulsions. An investigation was carried out to evaluate combined ultrasonication and HPH as an efficient technique for preparing stable oil-in-water nanoemulsions with reduced energy input [157]. Sequential application of ultrasonication and HPH at low to medium energy densities produced nanoemulsions with smaller droplet sizes and higher stability than those obtained using individual high-energy ultrasonication or HPH treatments. The results further indicated that ultrasonication prior to HPH was the most effective configuration, highlighting hybrid ultrasonication–HPH processing as a superior and energy-efficient nanoemulsion preparation technique. Thus, microfluidic homogenization and hybrid high-energy approaches can generate nanoemulsions with small droplet size and narrow PDI, improving stability and suitability for topical delivery [158]. However, superiority claims should be interpreted cautiously because performance is formulation-dependent and may be limited by heat and energy input, equipment cost, and instability during thickening with gelling agents arising from pH, electrolytes, and polymer–surfactant interactions.

High-energy methods offer advantages such as good control over droplet size, narrow size distribution, and scalability, making them suitable for pharmaceutical applications, including oral, parenteral, and topical drug delivery systems. However, they may have limitations such as high energy consumption, equipment cost, and potential thermal or mechanical degradation of heat-sensitive drugs. Overall, high-energy methods are robust and reliable approaches for producing pharmaceutically acceptable nanoemulsions with enhanced stability and bioavailability. Table 3 provides an overview of commonly used high-energy methods for nanoemulsion preparation.

### 6.2. Low Energy Methods

Low-energy emulsification methods are widely employed in the preparation of nanoemulsions intended for cutaneous drug delivery systems due to their simplicity, low cost, and avoidance of sophisticated high-shear equipment. Unlike high-energy methods, these techniques rely on the intrinsic physicochemical properties of the system, such as interfacial tension and phase behavior, to generate nanosized droplets. Such approaches are particularly advantageous for thermolabile drugs, bioactives, and cosmetic–pharmaceutical formulations. Frequently used low-energy methods include phase inversion composition (PIC), PIT, spontaneous emulsification, self-emulsifying drug delivery systems (SEDDS), DPE, and aqueous titration method.

#### 6.2.1. Phase Inversion Temperature

PIT method exploits the temperature-dependent solubility of surfactants, wherein oil, water, and the surfactant are heated with continuous stirring until the PIT is reached, followed by rapid cooling (e.g., ice-bath quenching) to form nanoemulsions [163]. Nanoemulsions prepared by the PIT method typically employ non-ionic surfactants containing temperature-sensitive polyoxyethylene chains, whose hydration–dehydration behavior governs phase inversion. The hydrophilic–lipophilic behavior of nonionic surfactants is strongly temperature dependent due to changes in the hydration of their poly(oxyethylene) chains. At low temperatures, these surfactants exhibit positive spontaneous curvature, leading to the formation of direct (oil-in-water) structures, whereas at high temperatures, dehydration of the ethoxylate chains results in negative spontaneous curvature and reverse (water-in-oil) structures. At an intermediate temperature, known as the HLB or PIT, the spontaneous curvature approaches zero, promoting the formation of bicontinuous microemulsions or lamellar liquid crystalline phases. At this temperature, ultra-low interfacial tensions are achieved, which significantly enhance emulsification efficiency [164]. Recent evidence shows that surfactants containing short polyoxypropylene chains can also be effective, as their temperature-dependent hydration enables phase inversion [165]. Polyoxypropylene-based surfactants with approximately 2.5–6.1 units produce stable oil-in-water nanoemulsions with spherical droplets in the 20–300 nm range, highlighting the importance of surfactant molecular architecture in PIT-based nanoemulsion formation. It is worthwhile to note that significant non-linear PIT behavior was observed with commercial ethoxylate surfactants, deviating from the assumed linear PIT-composition relationship [166]. This non-linearity, attributed to oil-like components within the surfactants, highlights the limitations of PIT-based linear mixing assumptions and underscores the need to refine hydrophilic–lipophilic deviation predictions for complex commercial surfactant systems. The type of emulsion obtained depends on the relationship between the PIT and the intended storage temperature of the final product, commonly 25 °C for room-temperature formulations. Systems prepared close to the PIT are thermodynamically sensitive; therefore, stability can be improved by incorporating cosurfactants or by adjusting the PIT using inorganic salts [167]. Complete solubilization of the oil phase within a bicontinuous microemulsion during PIT processing results in oil-in-water nanoemulsions with small droplet size and low PDI compared to PIC method [168]. A benidipine-loaded oil-in-water nanoemulsion was successfully developed using the PIT method and optimized by a Box–Behnken design [169]. Optimization of oil, surfactant, and glycerol concentrations produced a stable, transparent nanoemulsion with nanosized droplets (~97 nm), enhanced in vitro drug diffusion, and spherical morphology. Statistical analysis confirmed the significant influence and validity of all formulation variables, demonstrating the effectiveness and cost-efficiency of the PIT method for nanoemulsion development. Similarly, cajeput essential oil nanoemulsions were successfully prepared using the PIT method [170]. Optimization of surfactant type, concentration, oil content, and temperature identified a PIT of ~85 °C, with Tween 80 producing stable nanoemulsions containing up to 10% oil that remained physically stable for over 120 days. PIT-based nanoemulsions are especially suitable for topical and transdermal applications due to their small droplet size, uniformity, and enhanced skin permeation, although careful temperature control is required. Its key limitation is strong temperature and formulation sensitivity since commercial surfactants can show non-linear PIT behavior [166]. In addition, products stored near PIT may be unstable unless PIT is shifted with cosurfactants/salts and tightly controlled.

#### 6.2.2. Phase Inversion Composition

In the PIC method, or emulsion inversion point method, nanoemulsions are formed by gradually changing the system composition, typically through controlled addition of the aqueous phase to a pre-mixed oil–surfactant mixture at constant temperature under gentle stirring [171]. This compositional variation alters the surfactant curvature from favoring water-in-oil to oil-in-water structures, passing through near-zero curvature intermediates such as bicontinuous microemulsions or lamellar phases, ultimately resulting in the formation of nanosized droplets. Numerous studies have associated nanoemulsion formation via the PIC method with phase transitions involving lamellar and/or bicontinuous phases [172]. The manner and duration of transition through these intermediate phases determine the efficiency of emulsification, ultimately influencing both droplet formation and the final size distribution of the nanoemulsion. During the transient stage, controlled water addition and adequate shear are required to ensure efficient droplet breakup, as inadequate mixing or improper residence time can result in polydisperse nanoemulsions [173,174]. Although this procedure appears similar to self-emulsification, the mechanisms differ fundamentally, as PIC involves a progressive change in surfactant spontaneous curvature, whereas self-emulsification occurs without curvature inversion. In contrast, surfactant-free self-emulsification phenomena, such as the Ouzo effect, generally yield nanoemulsions with low oil volume fractions, which can restrict their applicability in pharmaceutical formulations. PIC is particularly attractive for dermatological formulations as it can be performed at ambient temperature, minimizing drug degradation and allowing easy scale-up. Under optimized conditions, the PIC method produced well-dispersed, spherical nanodroplets with very small particle size and low PDI, demonstrating good kinetic stability over storage. This method is also referred to as catastrophic inversion, as it involves a progressive increase in the volume fraction of the dispersed phase. A composition-sensitive polyoxypropylene-based surfactant was employed as an emulsifier to prepare n-dodecane-in-water nanoemulsions via the PIC method [175]. Control of surfactant concentration, oil-to-surfactant ratio, and electrolyte content enabled effective control over droplet size and morphology. These findings demonstrated that the PIC method is a versatile and efficient strategy for producing stable nanoemulsions using composition-responsive surfactants, with strong potential for large-scale and industrial applications. Ginger oil-in-water nanoemulsions were prepared using the PIC method with Tween 80 as the emulsifier [176]. The influence of key processing parameters, including stirring rate and water addition rate, on nanoemulsion characteristics was systematically optimized using response surface methodology.

#### 6.2.3. Spontaneous Emulsification

The spontaneous emulsification or self-nanoemulsification method involves the formation of nanosized droplets when an oil phase containing a surfactant is mixed with an aqueous phase containing a cosurfactant [177]. The rapid diffusion of water-miscible components, such as the solvent, surfactant(s) from the oil phase into the aqueous phase generates intense interfacial disturbances, increasing the oil–water interfacial area. This leads to the spontaneous formation of fine oil droplets when the bicontinuous microemulsion phase disintegrates. Solvents promote nanoemulsion formation both in the presence and absence of surfactants; when surfactants are absent, the phenomenon is termed the ouzo effect [178]. The sequence of component addition does not critically affect the process, as nanoemulsion formation occurs spontaneously due to interfacial instabilities. Surfactant-free microemulsions form at specific compositional ratios within the “pre-ouzo” region [179]. Their dilution without phase transitions enables spontaneous nanoemulsification, a key industrial approach for producing nanoemulsions at a commercial scale. The energy required to form new droplets depends on the interfacial tension and the number and size of droplets formed; systems with lower interfacial tension require less energy to emulsify. In theory, only mild agitation is needed for spontaneous emulsification, as the chemical potential difference between the oil and water phases is sufficient to initiate droplet formation. However, in practice, some formulations require gentle heating or cooling to trigger phase inversion and facilitate emulsification, particularly in temperature-sensitive systems. Despite extensive investigation, the mechanism of spontaneous emulsification remains unclear, as emulsification has been observed even without surfactants, indicating that interfacial turbulence alone does not fully explain the process [180].

#### 6.2.4. Self-Emulsifying Drug Delivery Systems (SEDDS)

Owing to their simplicity, scalability, and cost-effectiveness, SEDDS are promising systems for topical and transdermal delivery as it enhances drug solubilization, skin hydration, and permeation, especially for poorly water-soluble drugs. For instance, poorly aqueous-soluble curcumin-loaded self-nanoemulsifying drug delivery systems (SNEDDS) were developed using black seed oil, medium-chain mono and diglycerides, and surfactants for transdermal delivery [181]. The optimized formulation produced nanosized droplets (~71 nm) with high drug loading (~45 mg/g) and, when incorporated into a gel, demonstrated significant anti-inflammatory activity, achieving approximately 80% reduction in carrageenan-induced paw edema, indicating enhanced transdermal penetration and therapeutic efficacy. SNEDDS formulated as transdermal films and patches have been shown to markedly improve drug bioavailability and skin permeation [182]. Studies demonstrated up to twofold increases in permeation flux, Cmax, and AUC for saquinavir and acyclovir-loaded SNEDDS films compared with conventional drug films [183,184]. For SEDDS/SNEDDS, outcomes vary by formulation and model, and high surfactant/co-solvent levels with possible instability after incorporation may reduce performance without careful optimization.

#### 6.2.5. D-Phase Emulsification

The DPE technique for nanoemulsion preparation typically employs a surfactant, water, and oil, with the unique incorporation of an alkyl polyol, which promotes the formation of oil-in-water nanoemulsions [185]. In comparison with other low-energy emulsification methods, DPE requires lower surfactant concentrations, shows minimal dependence on HLB values, eliminates the need for organic solvents, and exhibits reduced energy consumption, particularly when compared with the PIC method [186]. Furthermore, studies have demonstrated that the inclusion of a poorly soluble oil phase is consistent with the underlying principles of DPE and contributes to the formation of stable nanoemulsions [187]. It was disclosed that the distinct self-assembled architecture of the D phase, together with its strong affinity for the oil phase, is fundamental to effective nanoemulsion formation [188]. The ordered arrangement of surfactant molecules within the D phase promotes the breakup of the oil phase into fine droplets within the intermediate oil/D-phase system. This is followed by a rapid redistribution of surfactants to the oil–water interface, which is essential for stabilizing the droplets at the nanoscale and preventing their coalescence. Studies comparing DPE with HPH using a design of experiments approach demonstrated that, within an optimized design space, DPE can produce olive oil-based oil-in-water nanoemulsions with droplet sizes (~275 nm) comparable to those obtained by high-energy processes, highlighting its effectiveness and potential advantages in nanoemulsion development [189]. In a recent study, nanoemulsions prepared using D-phase emulsification with surfactin as a single bio-emulsifier exhibited uniform droplet sizes (100–200 nm), excellent thermal stability, and enhanced skin penetration of retinyl propionate [190]. These findings demonstrate that DPE is an efficient, low-energy, and environmentally friendly approach for developing stable nanoemulsion-based cosmetic delivery systems.

#### 6.2.6. Aqueous Titration Method

Aqueous titration is a low-energy, phase-transition method for preparing thermodynamically stable nanoemulsions, in which the aqueous phase is gradually added to a mixture of oil, surfactant, and cosurfactant under gentle stirring, resulting in progressive droplet size reduction due to decreased interfacial tension. Pseudoternary phase diagrams are constructed to identify nanoemulsion regions, and formulation characteristics such as globule size, clarity, and stability are optimized by adjusting component ratios to ensure long-term thermodynamic stability [191,192]. The main limitation of this approach is the need for extensive phase-diagram mapping and careful ratio (e.g., oil: surfactant; surfactant: cosurfactant) optimization, often requiring relatively high surfactant/cosurfactant levels to maintain long-term stability.

Overall, low-energy methods offer significant advantages in the development of topical and transdermal nanoemulsions, including reduced energy consumption, improved formulation stability, enhanced skin penetration, and patient-friendly processing conditions. These methods continue to gain importance in pharmaceutical and cosmeceutical research for designing effective and safe dermal drug delivery systems. Table 4 presents an overview of commonly used low-energy methods for nanoemulsion preparation.

## 7. Therapeutic Applications of Nanoemulgels in Topical Drug Delivery

Nanoemulgels have been extensively investigated for a wide range of therapeutic applications in topical drug delivery owing to their ability to combine enhanced drug solubilization, controlled release, and improved skin permeation with favourable application characteristics. Recent studies demonstrate that nanoemulgels are particularly advantageous for the delivery of lipophilic and poorly water-soluble drugs, where incorporation into the oil phase of the nanoemulsion improves drug loading and thermodynamic activity, while the gel matrix ensures adequate skin residence time and patient compliance. Consequently, nanoemulgels have emerged as promising delivery systems for both local dermal therapies depending on formulation composition and drug properties.

### 7.1. Wound Healing

Wound healing is a complex, multistage biological process involving inflammation, tissue proliferation, angiogenesis, and remodeling, and effective topical therapy requires sustained drug availability, optimal moisture balance, and protection of the wound environment [195]. Nanoemulgels have emerged as promising topical delivery systems for wound management due to their ability to integrate the penetration-enhancing properties of nanoemulsions with the favorable application and retention characteristics of hydrogels. In general, hydrogels are promising alternatives to conventional wound dressings because their three-dimensional, water-rich polymer network maintains a moist environment, enhances drug penetration across the wound surface, helps control pathogens, and thereby promotes effective burn wound healing [196].

Hyaluronic acid, a biocompatible extracellular matrix component, supports dermal regeneration and angiogenesis. Therefore, hyaluronan-based nanoemulgels offer dual benefits of enhanced drug delivery and tissue repair, and were investigated in this study to improve the wound-healing efficacy of a 1,2-disubstituted benzimidazole derivative in infected wounds [197]. The hyaluronan-based nanoemulgel of new benzimidazole analogue showed good physicochemical stability (~182 nm, PDI 0.32, zeta potential −33 mV) with stability up to three months, significantly enhanced skin drug deposition (>3-fold), and superior wound-healing efficacy compared with silver sulfadiazine, supported by reduced inflammatory and oxidative markers, increased collagen and VEGF expression, and accelerated tissue regeneration.

Normal wound healing typically completes within about 20 days; however, conditions such as infection, metabolic disorders, cancer, or excessive inflammation can impair this process, leading to chronic wounds, excessive exudate, and prolonged health and economic burdens [197]. The use of topical formulations containing anti-infective and anti-inflammatory agents, such as xanthones, can enhance healing and reduce the risk of chronic wound development. Because these products are applied directly to open wounds, ensuring sterility through appropriate sterilization methods is essential to prevent microbial contamination and support safe and effective wound management [198]. The sterilized xanthone-loaded nanoemulgel formulated with sodium alginate (5% *w*/*w*) and Pluronic F127 (3% *w*/*w*) provided optimal viscosity and retained bioactivity after sterilization, significantly enhancing fibroblast proliferation and migration. The formulation promoted the release of bFGF and KGF/FGF-7 from skin fibroblasts and accelerated re-epithelialization in mouse wound models, along with improved collagen deposition and inflammation suppression, demonstrating its strong potential for topical wound-healing applications.

Nanoemulsions encapsulating phytochemicals and subsequently dispersed within hydrogel matrices to form nanoemulgels represent an advanced strategy for topical wound therapy. Numerous studies have demonstrated the effectiveness of phytochemical-loaded nanoemulgels in wound healing, with formulations containing compounds such as curcumin [199], ferulic acid [200], and other polyphenols, either alone or in combination with synthetic drugs. These formulations have shown enhanced skin penetration, reduced inflammation and oxidative stress, accelerated re-epithelialization, increased collagen deposition, and overall faster wound closure in preclinical models. The herbal extracts also demonstrated significant wound closure effects, further supporting their potential as effective agents for topical wound-healing therapy. The ethanol and ethyl acetate extracts of Cuscuta chinensis loaded in nanoemulgel showed strong antioxidant and anti-inflammatory activity, significantly reducing pro-inflammatory cytokines, and markedly enhanced wound contraction compared to Fucidin cream [201].

Curcumin enhances wound healing by regulating haemostasis, reducing inflammation and oxidative stress, promoting fibroblast activity, collagen deposition, angiogenesis, and wound contraction across all healing phases. However, its clinical efficacy is limited by poor solubility and bioavailability, which can be overcome using nano-based delivery systems [202]. A novel chitosan–alginate–cyclodextrin–curcumin (CA-CDCur) nanoemulsion was developed to enhance the water solubility and pharmacological efficacy of curcumin [203]. The formulation significantly accelerated wound healing through its antioxidant and anti-inflammatory effects and TGF-β1 induction, demonstrating efficacy across all four stages of wound healing.

Burn injuries are complex wounds marked by a prolonged inflammatory phase that delays healing. To enhance therapeutic efficacy, a nanoemulsion-based gel system incorporating the nutraceuticals (curcumin and resveratrol) was developed and evaluated for burn healing [204]. The optimized nanoemulgels exhibited nanosized droplets (~167–180 nm), low PDI, and negative zeta potential, indicating good physicochemical stability. Ex vivo studies demonstrated high skin retention of the nutraceuticals, with approximately 60% of the applied dose retained after 48 h. In vivo evaluation in a rat burn model showed that the combined curcumin–resveratrol nanoemulgel significantly improved burn healing, as evidenced by reduced inflammation and oxidative stress, restoration of biochemical markers, and histopathological features comparable to normal skin.

Full-thickness impaired wounds represent a major global healthcare challenge, often leading to significant morbidity, limb amputation, and increased mortality among affected patients worldwide [205]. In recent years, increasing attention has been directed toward combinatorial strategies for wound healing that integrate nanotechnology with bioactive herbal compounds and extracellular matrix components to enhance therapeutic outcomes [205,206,207]. The nanoceria-enriched decellularized goat small intestine submucosa (DG-SIS/Ce/NC) nanoemulgel exhibited strong free radical-scavenging and antibacterial activities, along with sustained curcumin release (62.9% in 96 h) and high skin permeability up to 79.7% in 96 h [208]. It also promoted enhanced cell growth under both normal and oxidative stress conditions and achieved near-complete full-thickness wound closure (97.33% in 14 days), accompanied by markedly increased collagen deposition at the wound site (1.61 μg/mg in 14 days), demonstrating its strong potential for full-thickness wound-healing applications.

Wound infections require prompt management, as necrotic tissue promotes microbial colonization and subsequent complications. *Staphylococcus aureus* is the most common cause of skin and soft tissue infections and may lead to severe local or systemic involvement if untreated [209]. Simvastatin exhibits notable activity against both methicillin-sensitive and methicillin-resistant *Staphylococcus aureus* [210]. In addition, this statin enhances VEGF-mediated angiogenesis, reduces oxidative stress, improves microvascular and endothelial function, thereby accelerating wound healing. Simvastatin nanoemulsion-based gel was developed for infected wound therapy, and the optimized formulation was incorporated into a gel using Carbomer 934 [211]. The optimized nanoemulsion exhibited a droplet size of 75 nm, PDI of 0.3, and zeta potential of −29.4 mV, which were maintained after gel incorporation, with a viscosity of 11.12 Pa·s. Both the nanoemulsion and the nanoemulgel remained thermodynamically stable at 4 and 25 °C for 72 days and showed enhanced antibacterial activity against this Gram-positive bacterium, with an MIC of 15.52 µg/mL representing a twofold improvement over the drug solution. Histological evaluation in a mouse wound model confirmed the superior wound-healing efficacy of the nanoemulsion gel compared with other formulations. Collectively, these evidences show that nanoemulgels are not only conceptually advantageous but can be practically engineered as stable, bioactive topical systems that accelerate wound healing endpoints, supporting their feasibility for clinical use in wound management. Nanoemulgel formulations developed for various wound healing applications, including excision, burn, diabetic, and infected wound models, are summarized in Table 5.

### 7.2. Dermatological Disorders

Inflammation is a complex protective immune response triggered by harmful stimuli or tissue injury to eliminate the cause of damage and initiate healing. However, chronic inflammation contributes to the development of skin disorders such as atopic dermatitis, contact dermatitis, eczema, psoriasis, rosacea, and seborrheic dermatitis, as well as systemic diseases including rheumatoid arthritis and osteoarthritis [219]. These conditions reduce quality of life and create a significant socioeconomic burden, largely driven by pro-inflammatory cytokines such as IL-1, IL-6, IL-8, and TNF-α [220]. Conventional systemic anti-inflammatory therapies suffer from limitations such as low bioavailability, metabolic degradation, and toxicity, while topical delivery is hindered by the skin barrier; novel formulations like nanoemulgels help overcome these challenges by enhancing epidermal permeability and therapeutic efficacy.

An aceclofenac-loaded nanoemulgel formulated using triacetin as the oil phase, Tween 80 and Cremophor EL as surfactants, and Transcutol HP with PEG 400 and ethanol as cosurfactants produced nanosized droplets (141.1 ± 3.65 nm) with low PDI and a negative zeta potential [221]. The resulting aceclofenac nanoemulgel demonstrated superior skin permeation compared with a marketed formulation, highlighting its potential for enhanced topical drug delivery. A diclofenac nanoemulgel prepared with clove oil, Tween 20, and PEG 400 was converted into a hydrogel using Carbopol 980. The formulation showed nanosized droplets, favorable rheological properties, enhanced drug release, and superior anti-inflammatory and analgesic effects compared with conventional gels, indicating its potential for topical pain and inflammation management [222].

Phytochemicals, either alone or in combination with synthetic drugs, when incorporated into nanoemulgels enhance topical therapy, while the combination can produce synergistic therapeutic effects in inflammatory disorders by targeting multiple inflammatory pathways, enhancing efficacy, reducing required drug doses, and minimizing adverse effects. A brucine-loaded nanoemulgel was developed to overcome the poor aqueous solubility and systemic toxicity associated with oral brucine administration [223]. The nanoemulgel, formulated using sodium carboxymethyl cellulose as a gelling agent, exhibited superior physicochemical properties, enhanced drug release, and significantly improved skin permeation compared with conventional gel and emulgel systems. In vivo studies demonstrated markedly improved anti-inflammatory and anti-nociceptive activities, while skin irritation studies confirmed good topical tolerability.

Besides anti-inflammatory effects, many phytochemicals incorporated into nanoemulgels provide additional therapeutic benefits such as antioxidant, antimicrobial, and anticancer activities. A nanoemulgel formulation of *Vitis vinifera* oil was formulated using a self-nanoemulsifying approach with Tween 80 and Span 80, followed by gelation with Carbopol [224]. The nanoemulgel exhibited nanosized droplets (<200 nm), low PDI, high physical stability (zeta potential < −35 mV), and pseudoplastic rheological behavior. Compared with the oil alone, the nanoemulgel demonstrated significantly improved antimicrobial activity against selected pathogenic strains, enhanced cytotoxic effects against multiple cancer cell lines, and effective inhibition of inflammatory enzymes, showing selectivity toward COX-1. A previous study by the same author developed a nanoemulgel using the same approach, incorporating *Capparis spinosa* oil [225]. The optimized nanoemulgel exhibited nanosized droplets (<200 nm), low PDI, high physical stability (zeta potential < −35 mV), and pseudoplastic rheological behavior. Compared with the native oil, the nanoemulgel demonstrated significantly enhanced antimicrobial activity against resistant bacterial strains, improved cytotoxic effects against multiple cancer cell lines, and effective inhibition of inflammatory enzymes with greater selectivity toward COX-1. A study reported the combination of eucalyptol and meloxicam and evaluated its potential to enhance anti-inflammatory efficacy when co-formulated in a topical nanoemulgel [226]. The formulation exhibited good stability, sustained drug release (6 h), enhanced skin permeation, and significant anti-inflammatory activity, supporting the synergistic potential of both components in the management of inflammation.

Psoriasis is a chronic, T-lymphocyte-mediated autoimmune inflammatory disease affecting the skin, joints, and tendons. This condition is characterized by moisture and lipid-deficient skin along with enhanced epidermal keratinocyte hyperproliferation, which is associated with reduced p53 expression [227]. In such conditions, topical treatment with available tacrolimus ointment is often ineffective due to poor skin penetration caused by epidermal thickening and limited drug availability resulting from reduced moisture and lipid content, thereby restricting its therapeutic usefulness. An azelaic acid and vitamin E-based tacrolimus lipid nanoemulgel was developed to enhance topical treatment of plaque psoriasis by improving drug localization and efficacy [228]. The optimized formulation (262.6 ± 9.2 nm; PDI 0.251 ± 0.03) showed skin-compatible pH, controlled drug release (83.7% in 12 h), slow permeation (38.8% in 24 h), and improved dermal retention compared with marketed tacrolimus ointment. In nanoemulgel systems, vitamin E additionally acts as a lipophilic bioactive and formulation stabilizer, improving drug solubilization within the lipid phase and contributing to enhanced skin penetration and retention. In another investigation, methoxsalen–Babchi oil nanoemulgels were developed with nanoscale droplet sizes (51.3–146.7 nm), high entrapment efficiency (92.76–98.10%), and stable zeta potential values ranging from −28.1 to −54.89 mV [229]. The optimized nanoemulgel demonstrated enhanced ex vivo skin penetration and localized accumulation of methoxsalen compared with the plain gel, which was further supported by in vivo studies showing significant improvement in psoriasis-related hyperproliferative skin symptoms.

Vitiligo is a common acquired skin depigmentation disorder characterized by the selective loss of functional melanocytes, leading to the development of well-defined white patches on the skin. Berberine chloride (Brb) protects melanocytes from oxidative stress and enhances melanophore activity, supporting melanocyte survival and function. However, its topical efficacy is constrained by poor skin permeation resulting from unfavorable physicochemical properties (pKa 2.47 and 15.7; log P −1.5). A Brb nanoemulgel enriched with clove oil was developed with a nanosized droplet diameter (20.50 ± 0.10 nm; PDI 0.178 ± 0.022) to improve skin delivery [230]. The formulation significantly enhanced skin permeation (14.25 ± 6.82 μg/cm^2^/h) compared with conventional Brb gel (8.38 ± 1.99 μg/cm^2^/h). In an experimental vitiligo model, the developed nanogel markedly reduced inflammatory and signaling markers (IL-6, IFN-γ, TNF-α, JAK1, and JAK3), as confirmed by mRNA and protein expression studies, leading to attenuation of disease progression and promotion of repigmentation.

**Figure 3:**
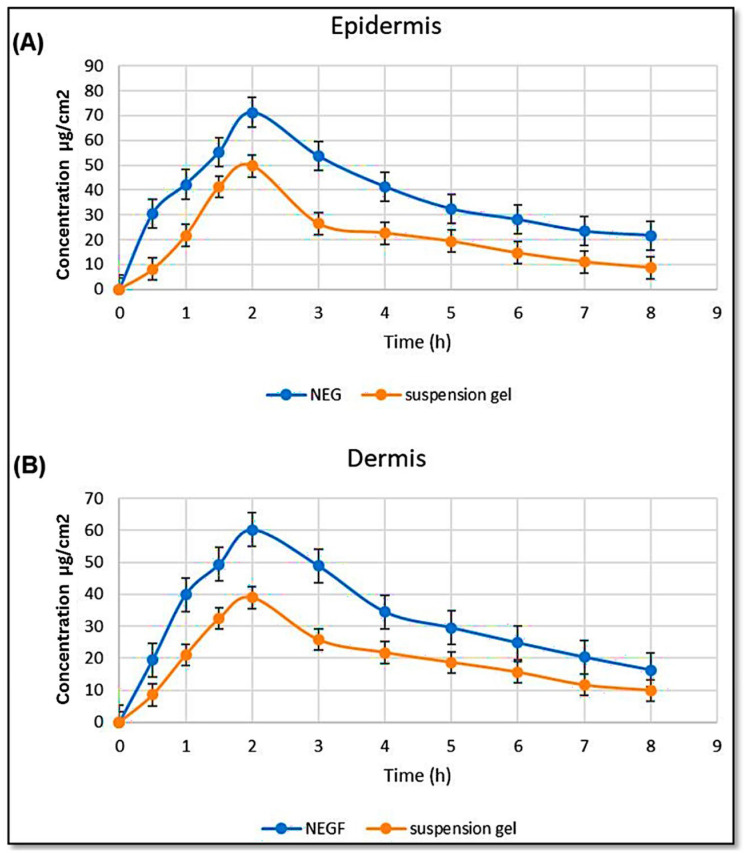
Concentration of *C. odorata* nanoemulgel in the (**A**) epidermis and (**B**) dermis. This figure is adapted from [235], used under a CC BY 4.0 license.

Atopic dermatitis is a chronic, relapsing inflammatory skin disease marked by severe pruritus, epidermal barrier dysfunction, and immune dysregulation [231]. Although conventional topical therapies such as corticosteroids and calcineurin inhibitors are effective, their prolonged use is often associated with adverse effects and poor patient adherence. Consequently, advanced topical drug-delivery systems, including nanoemulsions and nanoemulgels, have gained attention and offer a promising approach for the management of atopic dermatitis. Baricitinib, a Janus kinase (JAK1/2) inhibitor approved for atopic dermatitis in Europe, is associated with systemic adverse effects when administered orally. Baricitinib-loaded nanoemulgels were developed to enable topical treatment of atopic dermatitis while minimizing systemic adverse effects [232]. The formulations exhibited nanoscale globule sizes (162.86–173.66 nm), effective skin retention with predominant epidermal localization, and significant inhibition of inflammatory signaling pathways [p-STAT1 (*p* < 0.01) and p-STAT3 (*p* < 0.05)]. Histopathological and biochemical analyses confirmed reduced inflammation and disease severity, highlighting the potential of baricitinib nanoemulgel as an effective topical therapeutic strategy. Moisturizers are commonly used for the management of mild to moderate atopic dermatitis, and increasing attention has been given to phytochemicals due to their anti-inflammatory properties. Turmeric (*Curcuma longa*) has been widely explored not only in atopic eczema but also in psoriasis, owing to its potent immunomodulatory and anti-inflammatory activity. Incorporation of turmeric rhizome extract into a nanoemulgel formulation enhances cutaneous absorption and therapeutic performance [233]. In a mouse model, topical application of a 1% turmeric extract nanoemulgel significantly reduced key inflammatory mediators, including thymic stromal lymphopoietin, interleukin 13, and 17, while improving dermatitis scores and histopathological features (*p* < 0.05). A resveratrol-loaded nanoemulgel targeting the pro-inflammatory cytokine HMGB1 effectively reduced inflammation and improved skin barrier function in atopic dermatitis [234]. A *Cananga odorata* (ylang-ylang) essential oil-loaded nanoemulgel demonstrated enhanced efficacy in reducing scaling, erythema, and pruritus in scalp psoriasis and dandruff [235]. The nanoemulgel showed a significantly higher maximum skin concentration (Cskin max) in the epidermis (71.266 µg/cm^2^) compared with the conventional formulation (49.799 µg/cm^2^). Similarly, in the dermis, the nanoemulsion achieved a higher Cskin max (60.179 µg/cm^2^) than the conventional formulation (38.947 µg/cm^2^). These concentration profiles in the epidermis and dermis are illustrated in Figure 3. These findings highlight nanoemulgels as promising topical delivery systems for targeted, safe, and effective treatment of chronic inflammatory skin diseases. Table 6 summarizes nanoemulgel formulations for various dermatological disorders, highlighting the incorporated actives and key therapeutic outcomes.

### 7.3. Microbial Infections

Nanoemulgels have also gained significant attention in the treatment of fungal and microbial skin infections, where maintaining adequate drug concentration at the site of infection is critical. The enhanced retention and controlled release properties of nanoemulgels allow for prolonged drug availability on the skin surface and within the epidermal layers, leading to improved antifungal and antimicrobial activity. Additionally, the occlusive effect of the gel matrix increases skin hydration, which can further enhance drug diffusion and therapeutic outcomes in infected or damaged skin.

When incorporated into nanoemulgels, synthetic drugs or phytochemicals used either individually or in combination can show enhanced synergistic antimicrobial activity, along with additional therapeutic effects such as analgesic, anticancer, antidiabetic, and anti-inflammatory actions [242]. These benefits arise from improved drug solubilization, controlled and sustained release, increased skin permeability, enhanced bioavailability, and the ability to simultaneously modulate multiple biological and inflammatory pathways. The optimized clotrimazole nanoemulgel formulation exhibited suitable viscosity (1134.8 cP at 100 rpm), nanoscale globule size (355.3 nm; PDI, 0.464), negative zeta potential (−11.5 mV), high drug content (93 ± 0.28%), skin-compatible pH (6.21), and refractive index value, 1.433 [243]. Ex vivo permeation studies demonstrated higher drug permeation (83.61%) and an improved enhancement factor (3.16) compared with the marketed clotrimazole formulation (2.61). The clotrimazole nanoemulgel showed 2.42-fold higher in vitro antifungal activity against *Aspergillus niger* and caused no skin irritation in Wistar albino rats.

Nanoemulsions have shown superior dermal delivery of miconazole nitrate, demonstrating significantly enhanced antifungal efficacy against *Candida albicans* compared with conventional creams, thereby supporting their potential as effective topical and vaginal drug delivery systems [244]. In this context, an optimized self-emulsifying nanoemulsion of miconazole nitrate incorporated into a Carbopol hydrogel showed the highest drug release (41.8 mg/mL after 2 h) at 0.4% Carbopol [245]. Compared with a commercial cream, the nanoemulgel exhibited higher cumulative drug release (29.67% vs. 23.79% after 6 h) and significantly greater antifungal activity against *Candida albicans* (zone of inhibition: 40.9 ± 2.3 mm vs. 25.4 ± 2.7 mm), demonstrating the potential of nanoemulgel systems to enhance topical antifungal therapy.

Natural essential oils act as skin permeation enhancers in topical formulations, improving drug penetration while enhancing safety and therapeutic efficacy. Many essential oils such as *Thymus vulgaris*, *Cryptocarya aschersoniana*, *Cinnamomum amoenum*, *Allium sativum*, *Ligusticum chuanxiong* can act synergistically with antifungal agents by improving skin permeability, scavenging free radicals, and enhancing anti-inflammatory activity. The optimized cinnamon oil nanoemulsion, formulated using Pluracare L44 and Plurol Oleique CC 497 as surfactants and Capryol as a co-surfactant, showed an optimal globule size (92 ± 3 nm), high stability index (95 ± 2%), and a notable zone of inhibition (23 ± 1.5 mm) against *S. mutans* strain (ATCC 25175) [246]. Ketoconazole nanoemulgels prepared using clove oil (15%) or eucalyptus oil (20%) and gelled with Carbopol 943 and hydroxypropyl methylcellulose demonstrated enhanced drug solubility, permeability, and antifungal efficacy against *Candida albicans* [247]. The optimized formulations showed nanosized droplets (<100 nm) with low PDIs (0.24 and 0.26), high drug content (98.5 ± 2.2% and 98.8 ± 3.4%), and sustained drug release after 24 h (91 ± 4.5% and 89 ± 7%). Skin permeation was significantly improved (117 ± 7 and 108.34 ± 6 μg/cm^2^), and both formulations exhibited greater fungal growth inhibition than a marketed product without causing skin irritation, indicating their potential as safe and effective topical treatments for candidiasis.

Herbal therapies utilize a wide range of plant materials, including fruits, seeds, roots, bark, leaves, flowers, and, in some cases, the whole plant. Curcumin, similar to any other antibiotics uses the same method like membrane disruption, reactive oxygen species induction, efflux pump inhibition and cell division interruption to kill microorganisms [248]. The development and optimization of a curcumin-loaded nanoemulgel using linseed oil, vitamin E, and Carbopol 934 for antimicrobial and antifungal applications with minimal cytotoxicity has been reported [249]. The optimized formulation exhibited a particle size of (1121 nm), a zeta potential of (−29.2 mV), pH values (6.2–6.8), good spreadability (11.25–15.85), and suitable viscosity (1145.62–2258.47 cps). The optimized formulation demonstrated strong, dose-dependent antimicrobial activity against *Staphylococcus aureus*, *E. coli*, and *Salmonella*, and enhanced antifungal activity against *Aspergillus oryzae* and *Aspergillus niger* compared to curcumin alone. Cytotoxicity assessment revealed lower toxicity for the nanoemulgel, with cell viability of (25.64%) compared to pure curcumin (39.67%). Network pharmacology analysis suggested therapeutic action through inhibition of tubulin proteins, modulation of RNA translation, and regulation of cell signaling pathways, highlighting the formulation’s potential as an effective and safe antimicrobial and antifungal topical system. Various parts of the pomegranate plant exhibit strong antioxidant, anti-inflammatory, and immunomodulatory properties, contributing to a wide range of therapeutic activities [250]. Punica granatum seed oil-loaded nanoemulsion was prepared using the self-nanoemulsifying technique, with Span 80 and Tween 80 as emulsifiers, and was subsequently incorporated into a Carbopol hydrogel to obtain the nanoemulgel [251]. The nanoemulgel formulation demonstrated significant antimicrobial activity against MRSA, *Klebsiella pneumoniae*, and *Candida albicans*, with inhibition zones of 29 ± 1.1 mm, 26 ± 1.8 mm, and 18 ± 0.7 mm, respectively. It also exhibited enhanced cytotoxic activity against LX-2, B16-F1, Hep-3B, and HeLa cancer cell lines, with IC_50_ values of 169.82 ± 2.7, 39.81 ± 0.8, 61.65 ± 1.2, and 25.11 ± 1.3 μg/mL, outperforming the native oil. Additionally, the formulation showed anti-inflammatory activity through inhibition of both COX-1 and COX-2, with greater selectivity toward COX-1. Overall, the developed *P. granatum* seed oil nanoemulgel represents a promising pharmaceutical dosage form with improved therapeutic potential.

Recent studies have highlighted the effectiveness of nanoemulgel formulations in enhancing the therapeutic potential of plant-derived oils. Safrole oil nanoemulgel demonstrated significantly improved antibacterial, antifungal, antioxidant, antidiabetic, and anticancer activities compared with the native oil [242]. Similarly, Capsicum annuum oleoresin nanoemulgel exhibited notable antimicrobial and anticancer effects in vitro [252]. In addition, rosemary (*Rosmarinus officinalis*) essential oil nanoemulgel showed enhanced anticancer, antimicrobial, and antioxidant activities [253], while Coriandrum sativum oil nanoemulgel displayed significant antimicrobial and anticancer efficacy [254]. More recently, turmeric/neem-based topical nanoemulgels displayed strong antimicrobial efficacy [255], and carvone nanoemulgel emerged as a novel strategy for targeting methicillin-resistant *Staphylococcus aureus* [256]. Additionally, a lemon peel extract-based nanoemulgel was successfully developed as a non-toxic, antimicrobial, alcohol-free hand sanitizer [257]. In a recent study, bergamot oil-loaded nanoemulgel demonstrated strong broad-spectrum antibacterial activity against both Gram-positive and Gram-negative bacteria, highlighting its potential as an effective nano-based delivery system for pharmaceutical and personal care applications requiring enhanced drug delivery and antimicrobial efficacy [258].

Local cutaneous leishmaniasis is a neglected tropical disease caused by *Leishmania* parasites and transmitted through the bite of infected sandflies, affecting millions of people worldwide. Current treatments are limited by toxicity, poor patient compliance, and inadequate drug penetration, highlighting the need for effective topical and targeted therapeutic approaches. An optimized curcumin-loaded formulation exhibited high stability with good encapsulation efficiency (85 ± 5.4%) and drug content (68 ± 3.2%), achieving rapid drug release (90% within 4 h), while its nanoemulgel provided sustained release up to 46% over 24 h [259]. The selected formulation demonstrated significant in vitro antileishmanial activity with acceptable cytocompatibility against NIH3T3 fibroblasts (IC_50_ 0.6202 mM). Furthermore, the nanoemulgel showed low skin permeation but markedly higher drug retention in both ex vivo and in vivo studies, along with reduced systemic exposure, highlighting its potential as an effective topical therapy for cutaneous leishmaniasis and other skin disorders. Nanoemulgels can improve antimicrobial and antifungal therapy by enhancing solubilization, skin retention, controlled release, and hydration-driven diffusion, leading to higher local efficacy. However, performance and tolerability are formulation–dependent, so stability after gel incorporation and safety must be verified alongside droplet size and PDI. Table 7 summarizes nanoemulgel-based formulations investigated for the management of topical microbial infections.

### 7.4. Skin Cancer

Skin cancer is one of the most prevalent malignancies worldwide and includes melanoma and non-melanoma skin cancers such as basal cell carcinoma and squamous cell carcinoma. In the United States and Europe, skin cancer is the most commonly diagnosed malignancy, with nonmelanoma skin cancers accounting for the majority of new cases each year [266]. Conventional treatment options include surgery, radiotherapy, and chemotherapy; however, topical chemotherapy is limited by poor skin penetration, systemic toxicity, and low patient compliance. Novel drug delivery systems such as nanoemulgels have emerged as promising approaches to overcome these limitations [267].

Many anticancer agents recommended for skin cancer do not possess desirable HLB. For example, 5-Fluorouracil, approved for the treatment of actinic keratosis and superficial basal cell carcinoma, is a highly hydrophilic drug (log P −0.89). Its limited ability to penetrate the hydrophobic SC results in poor efficacy against deeper skin lesions [268]. Consequently, frequent and higher dosing is required, which often leads to adverse effects such as skin irritation and inflammation. Imiquimod is a well-established topical therapy for premalignant and early-stage skin cancers; however, it exhibits poor permeation through the hydrophilic dermal layers due to its low aqueous solubility. In addition, interactions between the drug’s amine groups and anionic skin components further restrict skin penetration, leading to reduced therapeutic efficacy [269]. The proven clinical use provides a strong foundation for developing advanced topical drug delivery systems aimed at enhancing local bioavailability and expanding the therapeutic potential of existing anticancer agents. Furthermore, multidrug resistance significantly limits the effectiveness of skin cancer chemotherapy due to complex drug–tumour interactions [270]. Insufficient drug delivery to tumour cells further compromises efficacy, underscoring the importance of optimizing the maximum tolerated dose and improving skin penetration to enhance therapeutic outcomes.

Consequently, strategies that enhance skin penetration and drug delivery to tumour sites hold significant potential for improving therapeutic outcomes in skin cancer treatment. Nanoemulgels enhance topical anticancer efficacy through multiple complementary mechanisms, including modulation of SC lipids by surfactants, co-surfactants, and oils, which improves higher local concentration gradients at the tumour site. Collectively, these effects promote enhanced drug retention within skin layers while limiting systemic absorption, aligning with established principles of topical nanocarrier-based localized therapy for skin cancer.

Although melanoma constitutes only about 1% of all skin cancers, it is highly aggressive and responsible for a disproportionate number of skin cancer-related deaths. This contrast highlights the pressing need for the development of more effective and innovative therapeutic strategies [271]. Extensive evidence highlights the beneficial role of polyphenolic compounds in regulating physiological functions and in preventing chronic diseases associated with oxidative stress, including cancer, diabetes, cardiovascular diseases, and neurodegenerative disorders [272]. Chrysin is a bioactive phytoconstituent with diverse pharmacological activities, including anticancer effects, and is considered safe and biocompatible; however, its clinical application is limited by poor water solubility and low bioavailability. A chrysin-loaded nanoemulgel exhibited nanosized droplets with uniform distribution, appropriate viscosity, and good spreadability [273]. Ex vivo skin permeation studies demonstrated significantly enhanced percutaneous absorption and drug retention. In vitro cytotoxicity studies on A375 and SK-MEL-2 melanoma cell lines confirmed enhanced anticancer efficacy, enabling potential dose reduction, while biocompatibility testing on L929 cells established the safety of the nanoemulgel.

Brb exhibits a wide range of therapeutic activities, including notable anticancer effects against melanoma, breast cancer, gastric cancer, hepatocellular carcinoma, and colorectal cancer. Brb exhibits anticancer activity in melanoma by inhibiting key signalling pathways involved in cell proliferation and survival (B-RAF/MEK/ERK, PI3K/AKT, NF-κB), inducing apoptosis through mitochondrial dysfunction and oxidative stress, suppressing metastasis-related processes (epithelial-to-mesenchymal transition), and modulating inflammatory and immune responses (AMPK activation, COX-2 inhibition, Toll-like receptors, cytokine signalling) to enhance its antitumor effects [274]. A Brb-loaded nanoemulsion-based gel was developed to enhance its anticancer efficacy in skin cancer therapy [275]. The optimized Brb nanoemulsion (Brb-NE) showed a mean particle size of 191.6 nm and a zeta potential of −4.8 mV, with improved in vitro cytotoxicity compared to free Brb, as indicated by IC_50_ values of 255.36 µM (Brb) and 189.53 µM (Brb-NE). The Brb-NE was incorporated into a Carbopol 934 gel, exhibiting suitable rheological properties and controlled drug release profile. Confocal and dermatokinetic studies demonstrated enhanced skin penetration and higher drug deposition in the epidermal and dermal layers, while HET-CAM testing confirmed the formulation was non-irritant, supporting its potential as a topical herbal therapy for skin cancer.

Recently, phenyl-isoxazole carboxamide derivatives have been synthesized and evaluated as promising anticancer agents, with a particular focus on melanoma therapy [276]. Their cytotoxic potential was investigated in vitro against a panel of cancer cell lines, including the melanoma B16F1 line, along with normal cell lines to assess selectivity. To improve cellular uptake and therapeutic performance, the most active compound was formulated into a nanoemulgel using a self-emulsifying technique. Among the tested compounds, compound 2a exhibited broad-spectrum anticancer activity, whereas compound 2e showed remarkable potency against melanoma cells, surpassing the reference drug doxorubicin. Notably, nanoemulgel incorporation of compound 2e further enhanced its cytotoxic efficacy by markedly reducing the IC_50_ value. Overall, current studies indicate that nanoemulgels may improve topical skin cancer therapy by enhancing penetration and local retention of challenging drugs while reducing dosing frequency and systemic exposure. However, findings remain largely formulation-specific and preclinical; therefore, standardized critical quality attributes, rigorous safety evaluation, and benchmarking against approved topical therapies in clinically relevant models are required. An overview of nanoemulgel-based formulations investigated for the management of skin cancer is presented in Table 8.

## 8. In Vitro Characterization and Biological Screening Methods

For topical nanoemulsion and nanoemulgel formulations, comprehensive physicochemical, rheological, release, permeation, and stability evaluations are essential to establish quality, performance, and therapeutic relevance. Physicochemical characterization includes droplet size and size distribution, typically measured by dynamic light scattering or photon correlation spectroscopy, based on Brownian motion-induced light scattering, where a mean droplet size in the nanometer range (generally <500 nm) and a low PDI (<0.3) indicate good physical stability, enhanced skin penetration, and controlled drug release [59]. Zeta potential, measured using a Zetasizer through particle movement under an applied electric field, with values ≥ ±30 mV, reflects electrostatic stability, aggregation tendency, and shelf-life. pH determination using a digital pH meter ensures skin compatibility (pH 5–6.5), minimizing irritation while maintaining drug stability [283]. Dilution potential, assessed by diluting the formulation with the external phase and observing clarity, turbidity, phase separation, and particle size, confirms thermodynamic stability [239]. Percentage transmittance, measured at 650 nm using a colorimeter, provides an indirect assessment of optical clarity, homogeneity, and droplet size. Conductivity measurements, performed using a platinized electrode under controlled temperature conditions, help identify emulsion type, phase inversion, and stability. Surface morphology, visualized by transmission electron microscopy after negative staining, allows direct observation of droplet size, shape, uniformity, and aggregation behaviour [284]. Drug content, determined by dissolving a known quantity of formulation and analyzing via UV-Vis spectrophotometry or HPLC, ensures dose uniformity and formulation accuracy, while encapsulation efficiency, calculated after separating free drug by ultracentrifugation, ultrafiltration, or dialysis, reflects the proportion of drug successfully entrapped within the nanoemulsion droplets and the drug loading capacity [259]. In vitro release testing is a key quality assessment tool for semi-solid dosage forms, including nanoemulgels, as drug efficacy and safety depend on drug release. Recommended by regulatory agencies such as the FDA, release studies are commonly conducted using vertical diffusion cells or immersion cells with an appropriate membrane under controlled temperature (32 ± 1 °C) and sink conditions for topical products [285]. Rheological evaluation of nanoemulgels includes viscosity and flow behavior, measured using a viscometer or rotational rheometer under varying shear rates, which governs ease of application, skin retention, drug release, and physical stability [264]. Spreadability, assessed using a texture analyzer by measuring the force required to spread the formulation, ensures uniform application and patient compliance, whereas adhesiveness, determined from force–time curves during probe withdrawal, indicates the formulation’s ability to remain in contact with the skin and enhance residence time. Extrudability, evaluated by measuring the force required to expel the formulation from a collapsible tube, reflects user convenience and practical applicability. In vitro drug release studies, commonly performed using Franz diffusion cells with synthetic dialysis membranes, assess cumulative drug release, release kinetics, and mechanism, providing insight into drug availability and formulation optimization [215]. Ex vivo skin permeation studies, conducted using excised animal or human skin mounted on Franz diffusion cells, yield parameters such as cumulative amount permeated, steady-state flux, permeability coefficient, and lag time, which predict skin penetration efficiency and correlate with in vivo performance [286]. Dermatopharmacokinetic testing is extensively evaluated by regulatory authorities to assess the safety, efficacy, and bioequivalence of topical and semisolid dosage forms. Key pharmacokinetic parameters such as Cmax, Tmax, and AUC are commonly used to characterize drug penetration and retention within the skin [287]. Dermatopharmacokinetic evaluation of semisolid dosage forms commonly employs tape stripping to quantify drug levels in the SC, microdialysis to monitor unbound drug concentrations in deeper skin layers in real time. Skin retention or deposition studies, including tape stripping and drug extraction or confocal laser scanning microscopy with fluorescent probes, quantify drug retained within different skin layers and visualize penetration depth, supporting targeting efficiency, prolonged local action, and safety [197,259,288]. Stability studies of pharmaceutical nanoemulsions, as outlined in the International Council for Harmonisation guideline Q1A (R2), are essential to ensure product consistency, safety, and efficacy by assessing the maintenance of physical, chemical, microbiological, and functional attributes over time [289]. These studies involve exposing nanoemulsions to stress conditions such as accelerated and long-term temperature storage, controlled humidity, light exposure, freeze–thaw cycles, and centrifugation to evaluate thermal stability, photostability, and resistance to extreme environmental and mechanical stresses [290]. During formulation development, preliminary screening includes heating–cooling cycles, followed by centrifugation and freeze–thaw testing, with only stable formulations progressing to further evaluation [291]. Cutaneous microdialysis enables real-time, minimally invasive measurement of drug levels in the dermal extracellular fluid [292], while confocal Raman microscopy provides non-destructive, label-free visualization and quantification of drug penetration across skin layers [293]. MALDI mass spectrometry imaging allows high-resolution spatial mapping of drug distribution within skin tissues [294]. In addition, emerging approaches such as 3D microfluidic skin-on-chip systems for dynamic absorption studies and computational pharmacokinetic models for predicting dermal drug behavior are increasingly utilized [295]. The biological properties of nanoemulgel systems for topical therapy, including antimicrobial, anticancer, wound-healing, anti-inflammatory activities, and dermal safety, have been extensively evaluated using established in vitro and in vivo models (Table 9).

## 9. Translation Challenges

### 9.1. Patents and Clinical Trials

Recent patent activity highlights the growing interest in topical nanoemulgel formulations for a wide range of therapeutic applications (Table 10). As summarized in the table, patented nanoemulgels have been developed using both synthetic drugs (e.g., tofacitinib citrate, diacerein, minoxidil) and phytochemicals or essential oils (e.g., ginger oleoresin, guggul extract, cinnamon oil, oregano oil, quercetin, mentha, chamomile, and coconut oil) for indications such as rheumatoid arthritis, wound healing, alopecia, photoaging, pitted keratolysis, and breast cancer. These inventions primarily emphasize enhanced skin permeation, improved therapeutic efficacy, reduced dosing frequency, and minimized systemic adverse effects, with several formulations demonstrating promising in vitro and in vivo performance in preclinical models. Despite encouraging in vitro, in vivo, and patent-based evidence, the clinical translation of topical nanoemulgels remains limited due to several key challenges [297]. Key barriers include scale-up feasibility and manufacturing reproducibility, as nanoemulgel systems are highly sensitive to variations in composition, processing conditions, and energy input [298]. Ensuring long-term physicochemical stability, particularly with respect to droplet size, phase separation, and drug leakage, remains a major concern during storage and commercialization. Additionally, the complex multicomponent nature of nanoemulgels often incorporating surfactants, cosurfactants, polymers, and bioactive phytochemicals raises issues related to skin irritation, toxicity, and excipient compatibility, necessitating extensive safety evaluation. From a regulatory standpoint, the lack of harmonized guidelines and standardized characterization protocols for nano-based topical drug delivery systems complicates approval pathways [299]. Despite this substantial body of patent-driven and preclinical evidence, there are currently no registered clinical trials evaluating topical nanoemulgels loaded with synthetic drugs or phytochemicals in human subjects. This highlights a significant translational gap between innovation and clinical application. The gap is driven mainly by regulatory and CMC (Chemistry, Manufacturing, and Controls) requirements, including GMP-scalable and reproducible manufacturing, tight control of critical quality attributes (droplet size/PDI, rheology/viscosity, content uniformity), validated long-term stability, packaging compatibility, microbial limits/sterility and preservative effectiveness. In addition, comprehensive safety evaluation of surfactant/co-solvent systems on compromised skin and stronger clinical trial designs with appropriate comparators are needed to translate patented topical nanoemulgel technologies into clinically approved dermatological therapies.

### 9.2. Future Perspectives

Topical nanoemulgels represent a promising drug delivery platform that combines the superior solubilization and skin permeation capabilities of nanoemulsions with the prolonged residence time, patient acceptability, and ease of application offered by gel systems [300]. Future perspectives in this field are increasingly shifting from mere formulation feasibility toward robust clinical translation, driven by characterization-focused regulatory expectations for topical dosage forms [301]. Advances will center on establishing clear microstructure–performance relationships, where parameters such as droplet size distribution, internal phase volume, rheological behavior, and polymer network strength are systematically linked to drug release, dermal uptake, and local tissue exposure, enabling rational excipient selection and stronger Quality-by-Design justifications [79]. Next-generation nanoemulgels are expected to emphasize dermal targeting and skin-retention engineering, optimizing follicular delivery, controlled barrier modulation, and localized drug deposition while minimizing systemic exposure. The development of stimuli-responsive “smart” nanoemulgels, hybrid systems integrated with advanced wound-care platforms, and the use of biocompatible, low-irritancy, and greener excipients will further enhance therapeutic performance and safety [265,302]. Progress in human-relevant preclinical skin models, scalable manufacturing strategies, standardized characterization, and data-driven formulation approaches using design of experiment and artificial intelligence/machine learning will be critical for improving reproducibility and predictability [303].

Advanced 3D in vitro skin and skin-on-chip models are well established in dermatology but still lack full physiological complexity [304]. Future developments will focus on integrating immune and microbiome components, improving extracellular matrix mimicking materials, and adopting microfluidic and real-time analytical technologies to create more predictive, scalable, and human-relevant platforms, thereby reducing reliance on animal testing and supporting personalized dermatological research. Although clinical translation is emerging, it remains limited compared with the extensive preclinical and patent activity. This highlights the need for focused early-stage clinical studies, regulatory alignment, and robust manufacturing strategies to fully realize the clinical potential of topical nanoemulgels in localized anti-inflammatory therapy, wound management, follicular disorders, skin cancer, and other dermatological applications.

## 10. Conclusions

Topical nanoemulgels represent a promising drug delivery approach for various dermatological conditions, offering improved skin penetration, localized drug action, and better patient acceptability. Nanoemulgels are generally cost-competitive and patient-convenient because they use a familiar topical manufacturing approach that combines a nanoemulsion with a gel base. They also offer good spreadability and acceptability for repeated application while improving local delivery of poorly soluble actives [305]. Commercially, nanoemulgels have demonstrated superior performance over conventional gels, with reports of improved tolerability and the potential for comparable or better therapeutic effect at reduced drug concentration. An example is diclofenac sodium nanoemulgel, which has been reported to show higher in vitro drug release and significantly improved analgesic/anti-inflammatory activity compared with both conventional and marketed diclofenac gels in in vivo models [222]. In contrast, nanovesicles such as liposomes can offer strong encapsulation and controlled release but are often more complex to formulate and characterize and may be prone to leakage or instability [306]. These challenges, along with additional regulatory and quality requirements, can increase development and production costs. Polymeric micelles can be efficient solubilizers and are often described as easier to prepare and sterilize; however, stability issues and regulatory expectations remain key obstacles to producing reproducible clinical products, limiting their competitiveness [307]. Microneedle patches can be highly efficient because they bypass the stratum corneum and allow self-administration [308]. However, they often involve higher fabrication costs and face manufacturing scale-up and regulatory hurdles, which can reduce cost-competitiveness even when patient convenience is high.

Most nanoemulsion formulations rely on high-energy emulsification techniques to achieve nanoscale droplets, with oils and lipids selected based on biocompatibility and drug solubilization capacity. Commonly used excipients in nanoemulgel formulations include non-ionic surfactants such as Tween^®^ 80 and Cremophor^®^ RH 40; cosurfactants and solubilizers such as PEG 400; cosolvents and penetration enhancers such as Transcutol^®^; and gelling agents including Carbopol polymers, cellulose derivatives, Pluronic^®^ F127, and chitosan. Collectively, these components contribute to improved formulation stability, appropriate viscosity, bioadhesion, and enhanced skin permeation. Nanoemulgels formulated with these excipients consistently demonstrate favorable physicochemical properties, controlled drug release profiles, enhanced skin permeation and/or retention, and promising in vitro and in vivo therapeutic efficacy with acceptable safety profiles. Based on the collective evidence summarized in our review, nanoemulgels appear to have the highest likelihood of success in conditions where high local drug levels, occlusion/hydration, and prolonged residence improve outcomes with measurable topical endpoints particularly in infected wounds and superficial microbial infections [309]. They also show strong potential in dermatitis and mild-to-moderate psoriasis by improving solubilization and skin retention, which may enable lower doses or reduced application frequency when tolerability is optimized [310]. Conversely, nanoemulgels likely have the lowest chance of success for indications requiring deep or highly targeted delivery, particularly advanced or thick lesions (e.g., late-stage skin cancers and deeply infiltrative tumors). This is because passive topical delivery, even with penetration enhancers, often cannot reliably achieve therapeutic concentrations throughout deeper malignant tissue while maintaining acceptable irritation profiles [311]. While extensive preclinical studies and patent developments highlight their therapeutic potential, clinical translation remains limited due to challenges in stability, scale-up, regulatory approval, and the lack of human clinical data. Addressing these gaps through standardized characterization, quality-driven formulation strategies, and targeted clinical studies is essential to advance topical nanoemulgels toward successful clinical application in dermatology.

## Figures and Tables

**Table 1:** Comparative assessment of nanoemulsions and alternative nanocarriers in cutaneous drug delivery.

Parameter	Nanoemulsions	Polymeric Micelles	Polymeric Nanoparticles	Lipid Nanoparticles (e.g., Solid Lipid Nanoparticles, Nanostructured Lipid Carriers)	Vesicular Nanocarriers (e.g., Liposomes, Niosomes)
Internal structure	o/w (oil droplets in water) or w/o (water droplets in oil), stabilized by a surfactant film.	Core–shell nanostructures formed by self-assembly of amphiphilic block copolymers.	Solid or semi-solid polymer matrix.	Solid lipid core or mixed solid/liquid lipid core stabilized by emulsifiers.	Bilayer phospholipid or surfactant membrane enclosing an aqueous core.
Drug loading capacity	Generally high for lipophilic drugs due to solubilization in the oil phase and interfacial region; Moderate for hydrophilic drugs by solubilization in the aqueous phase or at the interface, or co-loaded using nanoemulgels.	High for poorly water-soluble drugs solubilized in the hydrophobic core; limited for hydrophilic drugs.	High for lipophilic drugs with affinity to the polymer matrix, but may require organic solvents and complex optimization. Limited for hydrophilic drugs unless specialized architectures (e.g., core–shell) are used.	Moderate for lipophilic drugs limited by drug solubility in the solid lipid and risk of drug expulsion during storage. Generally low for hydrophilic drugs since they show poor solubility in lipid matrix and predominantly present in outer phase.	Moderate; lipophilic drugs partition into the bilayer but loading is constrained by bilayer packing; High for hydrophilic drugs when entrapped in the aqueous core.
Skin permeation efficiency	Often superior: small droplet size, high interfacial area, and surfactant-mediated lipid disruption.	Moderate; improved solubilization enhances diffusion, but effective transdermal delivery often requires penetration enhancers.	Variable; may improve follicular targeting and local deposition, but is often less effective for rapid systemic transdermal delivery without additional enhancers.	Improved over conventional formulations; good occlusivity and lipid similarity to skin enhance penetration, but permeation may be slower than nanoemulsions.	Good for superficial deposition; deeper penetration and systemic delivery can be limited without deformable or ultra-flexible vesicle designs.
Role of surfactants/penetration enhancers	High surfactant content; surfactants and co-surfactants often act as intrinsic penetration enhancers, fluidizing stratum corneum lipids.	Amphiphilic polymers (e.g., poloxamers) stabilize micelles; penetration enhancement is limited without additional enhancers.	Stabilizers (e.g., PVA, poloxamers) mainly act as steric/electrostatic stabilizers; penetration enhancement usually requires co-administration of separate enhancers.	Emulsifiers stabilize the particles and may contribute to permeation, but enhancer levels are typically lower than in nanoemulsions.	Surfactants/phospholipids may interact with skin lipids, but their primary role is vesicle formation; enhancement depends strongly on composition.
Physical stability	Kinetically stable with low risk of creaming or coalescence when optimized; long-term stability demonstrated in many studies.	Thermodynamically stable above critical micelle concentration; dilution may cause disassembly.	Generally good colloidal stability, but risk of aggregation or burst release if storage conditions are not controlled.	Good stability when stored appropriately; however, polymorphic transitions in solid lipids can lead to drug expulsion.	Prone to aggregation, fusion, and leakage of entrapped drug; often require low temperature storage and cryoprotectants for long-term stability.
Ease of preparation and scale-up	Relatively easy; produced by high or low-energy emulsification techniques.	Relatively simple self-assembly processes; scale-up feasible but sensitive to dilution and temperature.	Preparation may require organic solvents, complex purification, and specialized equipment, which can complicate scale-up.	Prepared by high-pressure homogenization or microemulsion techniques, but control of lipid crystallinity and polymorphism adds complexity.	Preparation can be technically demanding; scale-up requires careful control of hydration, size reduction, and sterilization steps.
Rheology and patient acceptability	Low viscosity systems that can be easily converted to nanoemulgels; non-greasy, transparent or translucent, with pleasant sensory attributes.	Low-viscosity aqueous systems usually require gel incorporation for topical use.	Often, aqueous dispersions may need incorporation into gels or creams for acceptable topical use.	Require incorporation into semisolid bases for dermal application; sensory feel depends on the external phase.	Aqueous dispersions that may appear turbid or milky; usually formulated into creams/gels for improved handling.
Typical applications in dermal/transdermal delivery	Anti-inflammatory drugs, analgesics, corticosteroids, antifungals, antioxidants, anticancer agents, and cosmetic actives with enhanced permeation and bioavailability.	Solubilization and delivery of poorly water-soluble drugs, especially for localized dermal therapy.	Targeted or controlled release delivery, including acne therapy, wound healing, and local chemotherapy.	Improved dermal deposition and sustained release of lipophilic drugs; widely explored for anti-inflammatory and analgesic drugs.	Local dermal therapy (e.g., anti-psoriatic, anti-eczema) and cosmetic applications, with emphasis on superficial skin layers.

**Table 2:** Overview of oil phases used in nanoemulsion systems for dermal and transdermal drug delivery.

Description	Examples/Trade Name	Primary Function	Application (Dermal/Transdermal)
Medium-chain triglycerides	Miglyol^®^, Labrafac^®^ WL 1349	Drug solubilization, nanoemulsion stability	Dermal and transdermal
Isopropyl myristate	IPM	Penetration enhancement, spreadability	Penetration enhancement, improved spreadability, primarily transdermal
Oleic acid, linoleic acid	Oleic acid	Stratum corneum lipid disruption, permeation enhancement	Dermal and transdermal
Mineral oil	Mineral oil	Emollient, occlusive effect	Dermal
Ethyl oleate	Ethyl oleate	Solubilization, permeation enhancement	Dermal and transdermal
Triacetin	Triacetin	Co-solvent, oil phase modifier	Dermal and transdermal
Propylene glycol monocaprylate (Type I)	Capryol™ 90, Capmul^®^ PG-8	Solubilization, penetration enhancement	Transdermal
Propylene glycol dicaprylocaprate	Labrafac™ PG	Oil phase, solubilization	Dermal and transdermal
Oleoyl polyoxyl-6 glycerides	Labrafil^®^ M1944	Oil phase, self-emulsification	Dermal and transdermal
Propylene glycol monolaurate	Lauroglycol™	Penetration enhancement	Transdermal
Polyglyceryl-3 diisostearate	Plurol^®^ diisostearique, Lameform^®^ TGI	Oil phase/emulsifier	Interfacial stabilization, Dermal/transdermal
Polyglyceryl-3 dioleate	Plurol^®^ Oleique CC 497	Oil phase/emulsifier	Self-emulsification, stabilization, Dermal/transdermal
Natural oils: Olive oil, castor oil	Pharma Olive™ (SOPHIM), Kollisolv^®^ Castor Oil	Oil phase	Biocompatibility, emollient effect, primarily dermal
Essential oils: Eucalyptus oil, clove oil, peppermint oil, cinnamon oil	Pharma Eucalyptus Oil™, Pharma Peppermint Oil™, Pharma Cinnamon Oil™	Oil phase	Permeation enhancement, synergistic therapeutic effects, Transdermal (low concentration)

**Table 3:** An overview of frequently used high-energy methods for nanoemulsion preparation, summarizing their principles, key variables, advantages, and limitations.

Method	Principle	Key Process/ Formulation Variables	Advantages	Limitations	References
High-pressure homogenization (HPH)	Coarse pre-emulsion forced through a narrow valve at high pressure (50–5000 psi); droplet disruption via hydraulic shear, intense turbulence, and cavitation; typically requires multiple passes until nanoscale droplets form.	Number of pass cycles, applied pressure, and process temperature/emulsifier type and concentration, oil: water ratio, surfactant blend/HLB.	Narrow size distribution; robust and widely used across pharma/food; good reproducibility and established industrial relevance.	High energy demand and long processing times; equipment wear at high pressure/many passes; batch processing may conflict with continuous industrial needs; potential temperature rise affecting product quality; sustainability/cost concerns.	[159]
Ultrasonic homogenization (Ultrasonication/ultrasound-assisted emulsification)	High-frequency ultrasound (~20 kHz to 100 MHz) via probe induces acoustic cavitation; microbubble formation/collapse generates local shear and shock waves that disrupt droplets; described via interfacial instabilities and cavitation dynamics.	Frequency, sonication time, power/amplitude, probe tip surface area; temperature control (cooling)/oil and aqueous phase composition; oil concentration and surfactant concentration strongly affect coalescence during processing; can use proteins/polysaccharides as stabilizers.	Easy lab operation; often lower surfactant requirement, reduced contamination risk; can achieve small droplets and narrow distributions; cleaning/control can be straightforward.	Scale-up constraints (probe geometry/energy distribution); minimum batch volume; longer processing possible; tip erosion/contamination risk; thermal effects can reduce emulsifier performance (increasing recoalescence).	[160]
Rotor–stator emulsification (RSE)/high-speed homogenization; colloid mill variants	Technique involves a rotating rotor (up to ~6600 rpm) equipped with teeth or blades and a stationary stator, forming a narrow high-shear gap that generates intense local shear forces and turbulence to promote effective droplet breakup. The process can be operated in batch (quasi-continuous) or continuous modes, such as in colloid mills, and is particularly suitable for emulsions with moderate to high viscosities (20 to 8000 mPa·s).	Rotor speed, processing time, stator geometry/gap; retention time in dispersing zone/Dispersed phase fraction; viscosity ratio and continuous phase viscosity; emulsifier adsorption rate; emulsifier type/concentration.	Lower capital cost than high-pressure systems; straightforward installation; compatible with viscous systems; handles larger volumes; useful as pre-emulsification for HPH.	Alone may require careful optimization to reliably hit nano-range for all formulations; droplet size depends strongly on viscosity and emulsifier kinetics; may yield broader distributions vs. microfluidization/HPH depending on setup.	[158]
High-pressure microfluidic homogenization (Microfluidization)	A coarse emulsion is driven by a high-pressure pump (30–120 MPa) into an interaction chamber (Y- or Z-type), where opposing high-velocity fluid streams collide within microchannels (~75 μm), leading to droplet disruption through intense shear, impact, and cavitation forces. Equipment often incorporates heat exchangers or cooling coils to dissipate excess thermal energy and ensure the formation of nanoemulsions with homogeneous droplet size distributions.	Operating pressure, number of passes, interaction chamber type (Y/Z), cooling efficiency, flow rate ratio and total flow rate/oil fraction; surfactant blend/HLB; protein stabilizers (e.g., whey) and their thermal sensitivity.	Efficient droplet size reduction; can be continuous and reproducible; strong control through chamber design and flow parameters.	Equipment cost; heat generation requires effective cooling; formulation-dependent limits (protein denaturation at high temperature; destabilization at >60 °C; still energy-intensive).	[161]
Hybrid/combined high-energy sequences (e.g., ultrasonic + HPH; high-speed mixing → HPH)	Uses a pre-emulsification step (high-speed rotor–stator mixing or ultrasonication) followed by high-pressure homogenization (or vice versa) to achieve smaller droplets at system-specific low to medium energy levels, resulting in reduced droplet size and improved stability compared to single-method processing.	Order of unit operations (ultrasonic → HPH vs. HPH → ultrasonic), energy density at each step, temperature/surfactant blend/HLB; oil fraction; surfactant: oil ratio; lipid: aqueous ratio.	Reduced energy requirement; improved droplet size/stability synergy; practical route to improve scalability and cost-effectiveness.	Requires more complex process control and validation; may increase processing steps/time if not optimized; still needs robust cooling/temperature control.	[162]

**Table 4:** An overview of low-energy nanoemulsion preparation methods and their key highlights.

Method	Principle	Key Process/ Formulation Variables	Advantages	Limitations	References
Phase inversion composition (PIC)	Emulsion inversion induced by composition changes (e.g., water content, surfactant curvature), leading to W/O → O/W transition via zero-curvature intermediates such as bicontinuous microemulsions or lamellar liquid crystalline phases.	Surfactant type and concentration; oil–water ratio; rate of aqueous phase addition; shear intensity; cosurfactant/co-solvent presence; pH for surface charge modulation; inversion kinetics.	Ambient-temperature processing; good droplet size control; enables zeta potential adjustment; scalable; suitable for thermolabile drugs and topical/transdermal delivery.	Sensitive to emulsification kinetics; transient viscous phases require slow addition and sufficient shear; may produce broad or bimodal size distributions; possible formation of polydisperse droplets.	[193]
Phase inversion temperature (PIT)	Emulsion inversion driven by temperature-dependent surfactant HLB and curvature changes; interfacial tension reaches a minimum at PIT, enabling nano-droplet formation upon rapid cooling.	Type of non-ionic surfactant; PIT value; heating–cooling rate; oil composition; cosurfactant/salt addition; storage temperature relative to PIT.	Produces nanoemulsions with small droplet size (20–100 nm) and low PDI (<0.2); rapid and reproducible; high emulsification efficiency.	Heating may cause thermal degradation or loss of volatile actives; temperature-sensitive stability; requires precise PIT control.	[119]
Spontaneous emulsification	Diffusion-driven interfacial instability occurs when an oil phase containing surfactant/cosurfactant comes into contact with the aqueous phase, causing spontaneous droplet formation without external energy.	Oil lipophilicity; surfactant and cosurfactant type and ratio (Smix); rate and mode of aqueous phase addition; solvent diffusion rate; stirring intensity; aqueous phase composition.	Simple and energy-efficient; no heating required; ideal for thermolabile drugs; high clarity; enhanced drug solubilization and skin permeation; suitable for topical and transdermal systems.	Often requires high surfactant concentrations; limited control over long-term kinetic stability; phase behavior must be carefully optimized; potential for irritation in dermal applications.	[59]
Self-Emulsifying/self-nanoemulsifying drug delivery systems	Formation of nanoemulsions in situ upon contact with an aqueous phase due to spontaneous emulsification driven by interfacial turbulence and diffusion of surfactant/cosurfactant, without external energy input.	Oil type and lipophilicity; surfactant and cosurfactant selection and ratio (Smix); drug solubility in components; dilution medium; incorporation into gels, creams, films, or patches for dermal use.	Simple and energy-efficient; excellent solubilization of lipophilic drugs; enhanced skin permeation and drug retention; suitable for thermolabile drugs; easily incorporated into topical and transdermal dosage forms.	Often requires high surfactant concentrations, which may cause skin irritation; risk of drug precipitation upon dilution; formulation optimization and safety evaluation are critical for dermal use.	[194]

**Table 5:** Topical nanoemulgel formulations developed for various wound healing applications.

Disease/Disorder	Drug/Phytochemical	Oil/Surfactant–Cosurfactant/Gelling Agent	Highlights	Reference
Excision wound healing	Thymoquinone	Black seed oil/Kolliphor EL- Transcutol HP/Carbopol 940	Droplet size: <100 nm; PDI: <0.54—both significantly (*p* < 0.05) influenced by ultrasonication time, irrespective of surfactant/co-surfactant ratio or concentration. Optimized nanoemulgel showed enhanced skin penetration and deposition. Exhibited pseudoplastic and thixotropic behavior, suitable for topical application. In preclinical wound models, it significantly accelerated wound healing, comparable to marketed silver sulfadiazine cream. Histopathology supported improved tissue regeneration.	[212]
Excision wound healing	Tunisian prickly pear (*Opuntia ficus-indica* L.) seed oil	Isopropyl myristate/Tween 80- Span 80/Sepimax Zen	Poor aqueous solubility was effectively improved by formulating the drug as a nanoemulsion. Nanoemulsion produced nanosized droplets (56.46 ± 1.12 nm) with narrow size distribution (PDI = 0.23 ± 0.01) and good physicochemical stability (zeta potential = −31.4 ± 1.4 mV). Nanoemulgel resulted in significantly improved wound healing compared with a marketed medicinal cream.	[213]
Microbial wound healing	Tea tree oil with mupirocin	Tea tree oil/Tween 80-PEG 400/Sodium alginate	Demonstrated suitable physicochemical properties for topical use, including skin-compatible pH (6.13). Nanodroplets size: 125.0 ± 3.6 nm with uniform size distribution, PDI = 0.204 ± 0.27. Showed appropriate viscosity (19,990 cP) and good spreadability (48.8 mm). Provided sustained drug release over 6 h (51.4%). Maintained good stability over 3 months under different storage conditions. Tea tree oil-based nanoemulgel formulation exhibited significant antibacterial activity.	[214]
Excision wound healing	Eucalyptol	Black seed oil/Span 60-Tween 80/Carbopol 940	Optimized formulation (F5) exhibited good homogeneity, good spreadability, and skin-compatible pH (5–6). Produced nanosized droplets (139 ± 5.8 nm) with PDI = 0.423 and good physicochemical stability (zeta potential = −28.05 mV). Achieved high drug content (94.81%) and excipient compatibility. In vivo wound-healing showed complete wound contraction in the experimental group (100 ± 0.015%). Performance significantly exceeded the standard (98.17 ± 0.75%) and control (70.85 ± 0.83%) groups. Overall, F5 appears to be a stable and effective wound-healing alternative.	[215]
Cutaneous wound healing	Tea tree oil and Jojoba oil	Tea tree oil and Jojoba oil/Tween 80:Span 80/Carbopol 940	Optimized nanoemulgel showed nanosized droplets (105.4 nm) with a high negative zeta potential (−39.2 ± 2.1 mV). Demonstrated acceptable cytocompatibility (CC_50_ = 453.82 ± 3.87 µg/mL). Exhibited broad-spectrum antimicrobial activity, along with strong antioxidant and anti-inflammatory effects. Wound-healing assessment included oxidative stress markers (SOD, MDA), inflammatory cytokines (TNF-α, IL-1β), and histopathological evaluation. Test group showed significantly enhanced wound closure, reduced inflammation and oxidative stress, and improved tissue regeneration versus controls (*p* < 0.05).	[216]
Diabetic wound healing	Thymoquinone–Fisetin	Coconut oil/Tween 80/Carbopol 940	Optimized Thymoquinone–Fisetin-loaded nanoemulsion exhibited a mean droplet size of 135.0 ± 5.2 nm, with PDI = 0.192 ± 0.005 and a mean zeta potential of −10.2 ± 0.5 mV. Phytochemicals co-loaded nanoemulgel, evaluated in Wistar rats, produced significant wound contraction over 21 days. Showed reduced oxidative stress and inflammation. Demonstrated enhanced fibrosis and increased expression of VEGF and TGF-β1. Histopathology indicated improved tissue features/regeneration Co-loaded nanoemulgel showed superior wound-healing efficacy compared with formulations containing thymoquinone or fisetin alone.	[217]
Burn wound healing	Snakehead fish powder	Olive oil/Tween80-Propylene glycol/HPMC	Optimized nanoemulsion exhibited a small droplet size (98.6 ± 0.93 nm) with a narrow size distribution (PDI = 0.1 ± 0.20) and a high zeta potential (−57.5 ± 0.3 mV). Ex vivo permeation via snake skin showed higher drug permeation from nanoemulgel formulations, especially NEG3 (66.75 ± 1.03% in 3 h), compared with the marketed cream (49.80 ± 3.42%). All formulations were non-irritant to the skin. In vivo burn wound studies showed the nanoemulgel significantly enhanced wound healing vs. marketed formulation (*p* < 0.05).	[218]

**Table 6:** Overview of nanoemulgel formulations developed for diverse dermatological disorders.

Disease/Disorder	Drug/Phytochemical	Oil/Surfactant–Cosurfactant/Gelling Agent	Highlights	Reference
Anti-inflammatory and Analgesic effect	Etoricoxib	Eucalyptus oil/Tween 20-PEG 200/Carbopol 934	Etoricoxib nanoemulgel optimized via Box–Behnken design demonstrated nanosized droplets (179.6 ± 4.21 nm), uniform size distribution (PDI = 0.373 ± 0.02), and a zeta potential of −10.9 ± 1.01 mV. Demonstrated good stability and sustained drug release (85.3% at 12 h). Showed enhanced skin permeation (0.072 cm^−2^ h^−1^). Produced superior analgesic activity (47.09% at 60 min) compared with a conventional gel. Exhibited strong anti-inflammatory activity (78.4% inhibition at 8 h) compared with a conventional gel.	[236]
Rheumatoid arthritis	Diflunisal	Eucalyptus oil/Tween 80-Transcutol P	Diflunisal (DIF) loaded nanoemulsion: particle size 23.03 ± 0.02 nm, PDI = 0.06 ± 0.005, zeta potential = −11.20 ± 0.10 mV, and viscosity = 19.73 ± 0.60 cP. Solubility-enhanced diflunisal (DIF-IC)-loaded nanoemulsion: particle size 20.86 ± 1.00 nm, PDI = 0.07 ± 0.001, and zeta potential = −13.76 ± 0.05 mV. The DIF-IC nanoemulgel, particularly when formulated with xanthan gum, showed superior skin permeation and enhanced anti-inflammatory efficacy compared with DIF. Overall, the DIF-IC nanoemulgel highlights strong potential as an effective topical therapy for inflammatory conditions.	[237]
Anti-inflammatory effect	Curcumin	Myrr oil/Tween 80-Propylene glycol/NaCMC	Emulgel: particle size 1698 nm (PDI = 0.338), whereas the nanoemulgel had a mean droplet/particle size of ~130 nm (PDI = 0.235). After 12 h, cumulative drug release was 72.17 ± 3.76% (gel), 51.93 ± 3.81% (emulgel), and 62.0 ± 3.9% (nanoemulgel). Curcumin-loaded nanoemulgel showed enhanced skin permeation (108.6 ± 3.8 µg/cm^2^·h). Demonstrated superior anti-inflammatory activity with minimal swelling (26.6% at 12 h). Findings confirm the effectiveness of the nanoemulgel system and the synergistic action of myrrh oil and curcumin.	[238]
Arthritic disorder	Piroxicam	Arachis oil/lecithin Lipoid S100/Carbopol 934	Selected formulation exhibited a particle size of 310.20 ± 19.84 nm, uniform size distribution (0.15 ± 0.02), and a zeta potential of −15.74 ± 1.6 mV. In vitro release showed a biphasic pattern: rapid release in the first 2 h, followed by a sustained release phase. Compared with a commercial gel, the optimized nanoemulsion demonstrated 1.66-fold higher analgesic activity with double the duration of action. Achieved a higher Cmax (45.73 ± 9.95 ng/mL vs. 28.48 ± 6.44 ng/mL) and showed a 2.41-fold increase in bioavailability.	[239]
Psoriasis	Ursolic acid	Patent protected	Hybrid nanoemulgel-based systems (nanoemulgel–macroemulsion and nanoemulgel–oleogel) were developed as topical carriers for ursolic acid in psoriasis therapy. Formulations were stable, skin-compatible, and well-tolerated by HaCaT keratinocyte cells. Demonstrated suitable viscosity, spreadability, and adhesion for topical application. Provided slow, controlled drug release consistent with established kinetic models. Systems show potential as effective vehicles for delivering ursolic acid to psoriatic skin.	[240]
Atopic dermatitis	Crisaborole	Caproyl^®^ PGMC/Cremophore EL: Propylene glycol/Carbopol 934	Nanoemulgel was developed to overcome the poor aqueous solubility of the marketed ointment. Optimized formulation (NE 9) showed a small droplet size (15.45 ± 5.27 nm), low PDI (0.098), and a zeta potential of −17.9 ± 8.0 mV. Nanoemulgel achieved a threefold increase in ex vivo skin permeation. Demonstrated superior in vivo therapeutic efficacy in Balb/c mice.	[241]

**Table 7:** Summary of nanoemulgel-based formulations investigated for the management of topical microbial infections.

Disease/Disorder	Drug/Phytochemical	Oil/Surfactant–Cosurfactant/Gelling Agent	Highlights	Reference
Antibacterial property	Omeprazole	Olive oil/Tween 80-Span 80/Carbopol 940	Chosen formulation showed nanosize (369.7 ± 8.77 nm) with PDI = 0.316 and zeta potential = −15.3 ± 6.7 mV. Exhibited drug content = 90.92 ± 1.37% and entrapment efficiency = 78.23 ± 3.76%. Demonstrated satisfactory in vitro drug release (82.16%). Showed satisfactory ex vivo permeation (72.21 ± 1.71 µg/cm^2^). Displayed effective antibacterial activity with MIC = 1.25 mg/mL. Chitosan coating further enhanced antibacterial efficacy.	[260]
Antifungal effect	Luliconazole	Eucalyptus oil/Tween 20-PEG 200/Carbopol 934	Optimized nanoemulgel exhibited nanosized globules (17 ± 3.67 nm), uniform size distribution (PDI < 0.5) and acceptable stability (−9.53 ± 0.251 mV). Showed high drug content (99.06 ± 0.59%), suitable viscosity (9.26 ± 0.08 Pa·s), skin-compatible pH (5.65 ± 0.17), and refractive index (1.31 ± 0.08). Demonstrated significantly enhanced ex vivo skin permeation compared with a conventional gel. Caused no skin irritation or histopathological abnormalities after 48 h. Produced a significantly larger zone of inhibition (*p* < 0.05).	[261]
Antifungal effect	Sertaconazole	Oleic acid	Optimized nanoemulgel showed nanosized droplets (111 nm) with high entrapment efficiency (99.7%). Exhibited suitable viscosity (2682 ± 96.77 cP) and good spreadability (7.03 ± 0.98 cm). In vitro studies demonstrated sustained drug release (77.00 ± 4.28%) over 8 h. Showed superior antifungal activity with larger zones of inhibition against Candida albicans (1.1 ± 0.1 cm) and Aspergillus niger (0.6 ± 0.1 cm) compared with marketed formulations.	[262]
Antibacterial and anti-inflammatory effects	Curcumin	Oleic acid/Tween 80 and Span80-PEG 400/Carbopol 940	Curcumin nanoemulsion showed nanodroplet size (12.26 nm) and low PDI (0.254). Curcumin nanoemulgel release profile followed the Higuchi diffusion-controlled drug release model. Demonstrated excellent antioxidant capacity (FRAP = Ferric Reducing Antioxidant Power; 533.49 ± 2.84 µM Fe (II)/g). Showed potential photoprotective efficacy (SPF = Sun Protection Factor; 13.86) and high anti-inflammatory activity. Demonstrated antibacterial activity against *Staphylococcus aureus* and *Escherichia coli*, with MIC = Minimum Inhibitory Concentration in the range 250 µg/mL ≤ MIC < 500 µg/mL.	[263]
Antibacterial	Mupirocin	Eucalyptus oil/Tween80-Span 80/Carbopol 940	Two mupirocin (MUP) nanoemulgels were prepared: MUP-NEG EO (eucalyptus oil–based) and MUP-NEG EU (eucalyptol–based). Particle size: MUP-NEG EU (~2500 nm) was significantly larger than MUP-NEG EO (~1000 nm) (*p* < 0.05). PD1: MUP-NEG EU (~0.51) was significantly higher than MUP-NEG EO (~0.50) (*p* < 0.05). Lower skin permeability than the marketed control, but higher mupirocin skin deposition at 8 h (no difference at 24 h), confirmed by micro-CT imaging. Antibacterial activity was comparable to the marketed control, despite nanoemulgels containing a lower drug amount, indicating formulation efficiency.	[264]
Antibacterial	Neomycin with silver/zinc oxide nanocomposite	Olive oil/Tween 80-PEG 400/Carbopol 940	Nanoemulgel formed a crystalline nanocomposite with a small particle size (38 nm). Achieved high drug loading (98%). Provided sustained drug release (81% in 12 h). Demonstrated excellent hemocompatibility. Showed strong antimicrobial and antibiofilm activity, particularly against *Staphylococcus aureus* (MIC = 0.002 µg/mL). Significantly improved wound healing in vivo vs. control (*p* < 0.05)	[265]

**Table 8:** Overview of nanoemulgel-based formulations investigated for the management of skin cancer.

Disease/Disorder	Drug/Phytochemical	Oil/Surfactant–Cosurfactant/Gelling Agent	Highlights	Reference
Skin melanoma	5-Fluorouracil	Isopropyl myristate/Span 80 and Tween 80-Transcutol P/Carbopol and glycyrrhizin	Optimized formulation had a nanodroplets size of 64.1 ± 5.13 nm and drug loading of 97.3 ± 5.83%. 5-Fluorouracil (5-FU)–loaded nanoemulgel (NEG) significantly improved ex vivo skin permeability up to 1440 min compared with plain 5-FU gel. In B16F10 melanoma cells, the formulations markedly reduced IC_50_: 20 µg/mL (plain 5-FU gel), 1.1 µg/mL (5-FU-NEG), and 0.1 µg/mL (GLY-5-FU-NEG). Demonstrated enhanced intracellular uptake (44.3–53.6%). Showed altered cell-cycle progression, supporting improved anticancer activity.	[277]
Skin carcinoma	Quercetin	Oleic acid/PEG 400-Tween 80/Carbopol 940	Developed quercetin-loaded nanoemulsion (QCT@NE) was nanometric in size (173.1 ± 1.2 nm), with low PDI (0.353 ± 0.13) and a zeta potential of −36.1 ± 5.9 mV. Showed good encapsulation efficiency (90.26%). Optimized quercetin-loaded nanoemulgel demonstrated markedly enhanced cytotoxicity against human skin carcinoma (A431) cells compared with plain quercetin. IC_50_ values: 108.5 µM (nanoemulgel) vs. 579.0 µM (plain quercetin). Skin irritation studies showed no signs of toxicity, supporting suitability for topical application. Hematological and biochemical evaluations showed no significant differences vs. controls, supporting systemic safety.	[278]
Squamous cell carcinoma	Quercetin	Clove oil/Tween 20: Propylene glycol/Carbopol 940	Optimized quercetin nanoemulsion (QUE NE3) exhibited good stability with a negative zeta potential (−38.2 mV), small particle size (98.2 nm), and high entrapment efficiency (96.36%). QUE NE3 loaded nanoemulgel showed significantly enhanced ex vivo skin permeability. Achieved 93.56 ± 1.16% cumulative drug release within 8 h, surpassing conventional quercetin gel and drug-free nanoemulgel. Confocal laser scanning microscopy (CLSM) confirmed dermal penetration. MTT assay showed dose-dependent anticancer activity against TE 354, A431, and A375 skin cancer cell lines. Highest cytotoxicity was observed against A431 cells (66.52%).	[279]
Skin melanoma	Daidzein	Ethyl oleate/Lipoid S100: Tween 80/Protasan ™ UP G 213	Nanoemulsion formulations showed smaller particle sizes (~150 nm) compared with nanoemulgel (NEG) systems (~190–200 nm). NEG formulations exhibited a positive zeta potential due to Protasan™ UP G 213. All formulations-maintained skin-compatible pH and followed zero-order release kinetics. Daidzein (DZ) release rate was higher from DZ-nanoemulsion (2.70 ± 0.27 µg/cm^2^/h) than from DZ-NEG (1.33 ± 0.12 µg/cm^2^/h). All formulations remained stable within the nano-size range. The systems preserved DZ cytotoxic activity against SK-MEL-30 melanoma cells and normal fibroblasts (PCS-201-012).	[280]
Skin melanoma and Psoriasis	Leflunomide	Capryol 90/Cremophor EL: Transcutol HP/Pluronic F127	Nanoemulsifying preconcentrate formed a stable nanoemulgel with a mean globule size (~123.7 nm) and viscosity (9620 ± 93 cP). Exhibited suitable mechanical properties: hardness (523 g), adhesiveness (431 g), and springiness (1.02). Ex vivo rat-skin permeation showed significantly enhanced flux, permeability, diffusion, and drug deposition due to nanoemulsification. In vitro cytotoxicity in HaCaT, A375, and SK-MEL-2 cell lines confirmed improved therapeutic efficacy.	[281]
Skin cancer	Imiquimod	Oleic acid-Rose oil/Tween 20: Propylene glycol/Carbopol Ultrez 10	Optimized nanoemulsion-based gel showed favorable physicochemical properties with small globule size (118 nm), high negative zeta potential (−56.26 mV), acceptable PDI (0.378), and excellent drug content (99.77%). Demonstrated improved in vitro drug release (45.00% in 8 h) compared with the commercial cream (34.32%). Showed significantly higher cytotoxicity against A431 cells (*p* < 0.001) with IC_50_ = 10.76 ± 2.54 µg/mL. In vivo, the formulation markedly reduced tumor incidence (16.66%), tumor volume (140.26 ± 3.48 mm^3^), tumor burden (5.50 mm^3^), and tumor mass (0.66 ± 0.05 g). Histopathology confirmed the absence of malignant skin changes.	[282]

**Table 9:** In vitro and in vivo biological evaluation of topical nanoemulgels.

Biological Property	Method/Instrument	Principle	Evaluation Parameters	Significance	Reference
Antimicrobial activity	Diffusion: Standardized inoculum is swabbed on agar (Mueller–Hinton agar for bacteria; Sabouraud dextrose agar for fungi). Wells/discs are loaded with formulation and controls, incubated (bacteria: 35–37 °C for ~18–24 h; fungi: ~25–30 °C for ~24–48 h or longer as needed), and zone diameter is measured. Minimum inhibitory concentration (MIC): Serial dilutions are prepared in broth (Mueller–Hinton broth/Sabouraud broth), inoculated, incubated as above, and MIC is recorded as the lowest concentration with no visible growth; Minimum bactericidal concentration (MBC)/Minimum fungicidal concentration (MFC) is confirmed by sub-culturing non-turbid wells onto agar.	Growth inhibition by formulation; diffusion gives inhibition zone, dilution gives minimum inhibitory/lethal concentration.	Zone of inhibition (mm); MIC; MBC (bacteria)/MFC (fungi).	Confirms antimicrobial potential against different types of microorganisms.	[224]
Anticancer efficacy and mechanisms	Respective cells are seeded in 96-well plates and incubated to allow attachment. The cells are treated with graded concentrations of the nanoemulsion/nanoemulgel for 24–72 h. Following treatment, MTT or SRB reagent is added and incubated to allow dye reduction or protein binding. The developed color is measured using a microplate reader, and cell viability (%) and IC_50_ values are calculated. Treated cells (near IC_50_ dose) are harvested. For apoptosis, cells are stained with Annexin V–Fluorescein Isothiocyanate/propidium iodide and analyzed by flow cytometry. For cell cycle, cells are fixed, stained with PI, and analyzed for DNA content.	Metabolically active cells reduce/bind dye; reduced signal indicates cytotoxicity Annexin V detects early apoptosis; propidium iodide detects late apoptosis/necrosis; DNA content analysis identifies cell-cycle arrest.	% cell viability, % growth inhibition, IC_50_ % apoptotic cells, % necrotic cells, cell-cycle phase distribution.	Screens anticancer potential and find mechanism of anticancer action.	[278]
Wound healing efficacy and potential	Appropriate in vivo wound model is created. The formulation is applied topically at regular intervals. Wound area is measured periodically using planimetry/image analysis until complete healing; tissue and microbial load may be analyzed when required. Histology (H&E, Masson’s trichrome); ELISA (for markers). Cells are seeded in culture plates and grown to a confluent monolayer. A straight “scratch” is made using a sterile pipette tip, and detached cells are removed by washing with buffer. Test and controls are added, and images are captured at 0 h and at predetermined intervals during incubation	Enhanced wound contraction, faster epithelialization, and tissue regeneration indicate improved healing. Migration and proliferation of cells into an artificial scratch simulate wound closure.	% wound contraction, epithelialization time, healing duration, VEGF, TGF-β, IL-6, TNF-α, IL-1β levels; histological features, % scratch closure, migration rate, time to closure.	Demonstrates accelerated tissue repair, antimicrobial benefit, reduced inflammation, and improved angiogenesis. Rapid screening of wound-healing potential at cellular level.	[199]
Skin irritation potential and safety/cytotoxicity	Draize: Formulation is applied to shaved animal skin and erythema/edema is scored at fixed intervals. Hen’s Egg Test-Chorioallantoic Membrane (HET-CAM): Formulation is applied to the chorioallantoic membrane of fertilized hen’s egg and vascular reactions are observed. Reconstructed Human Epidermis (RHE): Formulation is applied to reconstructed human epidermis; tissue viability and inflammatory markers are measured after exposure. HaCaT keratinocytes are seeded and grown to confluency, treated with graded concentrations of nanoemulsion/nanoemulgel, followed by addition of MTT/SRB/Alamar Blue reagent, and cell viability is quantified using a microplate reader.	Assesses inflammatory response and tissue damage following topical exposure; HET-CAM detects vascular irritation, RHE mimics human epidermis response. Viable epidermal cells metabolize or bind dye; reduced signal indicates cytotoxicity.	Dermal irritation-Erythema score, edema score (Draize); Vascular irritation-hemorrhage, lysis, coagulation time (HET-CAM); Tissue irritation-% cell viability, IL-1α release (RHE). % cell viability, % cytotoxicity, IC_50_, morphological changes.	Ensures dermal safety, supports regulatory acceptance, and confirms suitability for topical application. Early screening of dermal toxicity reduces animal testing.	[296]
Anti-inflammatory activity	In vitro-UV-Visible spectrophotometry for protein denaturation, nitric oxide, and antioxidant assays, ELISA for cytokine and COX enzyme quantification, cell-based assays such as MTT or Alamar Blue for cytotoxicity assessment. In vivo-Carrageenan-induced paw edema, histamine or formalin-induced inflammation, and croton oil or arachidonic acid–induced ear edema models, using tools such as plethysmometer, digital vernier calipers, ELISA kits, and histopathological microscopy.	Protein denaturation inhibition, HRBC membrane stabilization, nitric oxide inhibition, cyclooxygenase (COX-1/COX-2) enzyme inhibition, pro-inflammatory cytokine suppression (TNF-α, IL-1β, IL-6), antioxidant activity, and cell viability. The principle involves induction of inflammation followed by topical or systemic administration of the nanoformulation, where reduction in swelling, mediator release (PGE_2_, TNF-α, IL-1β), and tissue damage reflects anti-inflammatory efficacy.	Percentage inhibition of protein denaturation, membrane stabilization, nitric oxide and COX activity, cytokine concentrations in pg/mL, antioxidant scavenging percentage, and percentage cell viability. Percentage inhibition of paw edema or ear thickness, reduction in erythema and lesion score, changes in inflammatory biomarkers, and histopathological alterations.	Confirming the anti-inflammatory efficacy, safety, and therapeutic superiority of nanoemulsion/nanoemulgel systems through improved drug penetration, sustained release, and targeted delivery. Confirming in vitro findings, demonstrating pharmacodynamic effectiveness, skin retention, and safety of nanoemulsion/nanoemulgel systems under physiological conditions.	[251]

**Table 10:** Patent applications (https://www.wipo.int/portal/; accessed on 30 December 2025) related to topical nanoemulgel formulations.

Publication Number	Date of Publication	Title	Summary of Invention
2025/S/01208	26 February 2025	Cinnamon (Cinnamomum burmannii) essential oil nanoemulgel formula as a topical antiphotoaging preparation	The nanoemulsion comprises cinnamon essential oil, Cremophor RH40, and PEG 400 in a 1:8:1 ratio and is incorporated into a gel base at a 1:4 ratio. The gel base consists of CMC-Na (2%), glycerin (5%), propylene glycol (15%), methyl paraben (0.2%), propyl paraben (0.05%), and distilled water.
WO/2024/144693	4 July 2024	Unique nanoemulgel formulations developed from combinations of *Mentha spicata*, *Matricaria chamomilla* and *Cocos nucifera* oils and in vitro wound healing effects of the formulations	The invention describes a topical formulation combining *Mentha spicata*, *Matricaria chamomilla* essential oils, and *Cocos nucifera* oil in a suitable carrier, developed for wound healing applications.
202311053305	15 September 2023	Topical nanoemulgel formulation of tofacitinib citrate and diacerein	Topical nanoemulgel formulations of tofacitinib citrate and diacerein were developed to enhance skin delivery and reduce adverse effects associated with oral administration. Both formulations demonstrated significant anti-inflammatory activity in animal studies, with the diacerein nanoemulgel showing greater reduction in paw volume and the tofacitinib citrate nanoemulgel exhibiting a slightly greater reduction in paw diameter.
202221027485	17 June 2022	Nanoemulgel formulation of oregano oil and quercetin and the method of preparation thereof	Topical nanoemulgel formulation containing oregano oil and quercetin. The formulation demonstrates enhanced stability and bioavailability and is effective in the treatment of pitted keratolysis, while also helping to prevent its recurrence.
202021044492	22 April 2022	Topical nanoemulgel formulation for arthritic inflammation and pain	The invention involves an herbal topical nanoemulgel containing ginger oleoresin and lipid guggul extract. It improves patient compliance and reduces the frequency of dosage administration.
WO/2020/121329	18 June 2020	Minoxidil and castor oil nanoemulgel for alopecia	Topical nanoemulgel combining minoxidil and 10–30% castor oil for hair loss management. The formulation enhances hair density, diameter, and length and is prepared using a novel w/o/w emulsion-based process.
2018/S/00045	19 January 2018	Formula sediaan fitofarmaka nano emulgel dari fraksi etil asetat jahe penghambat sel kanker payudara t47d.	Phytopharmaceutical topical nanoemulgel formulated from the ethyl acetate fraction of ginger rhizomes inoculated with Arbuscular Mycorrhizal Fungi (AMF). The formulation is intended to inhibit T47D breast cancer cells, offering a plant-based therapeutic approach with improved efficacy and reduced side effects.

## Data Availability

No new data were created or analyzed in this study. Data sharing is not applicable to this article.
